# Supplementary material for: Estimated reduction in obesity prevalence and costs of a 20% and 30% ad valorem excise tax to sugar-sweetened beverages in Brazil: A modeling study
Source: PLoS Med. 2024 Jul 17;21(7):e1004399. doi: 10.1371/journal.pmed.1004399 (PMC11253955; doi:10.1371/journal.pmed.1004399)
Supplement: S1 Appendix — (PDF) [file pmed.1004399.s001.pdf]

S1 Appendix for "Expected reduction in obesity prevalence and costs of a 20% and 30% ad valorem excise tax to Sugar-Sweetened-Beverages in Brazil: a modeling study"

Ana Basto-Abreu, PhD[1], Rossana Torres-Alvarez, MSc[2], Tonatiuh Barrientos-Gutierrez, PhD[1], Paula Pereda, PhD[3], Ana Clara Durán, PhD[4], and Tonatiuh Barrientos-Gutiérrez, PhD *[1]

[1] *Center for Population Health Research. National Institute of Public Health, Cuernavaca, Mexico*

[2] *Department of Integrative Oncology, BC Cancer Research Institute, Vancouver, British Columbia, Canada School of Population and Public Health, University of British Columbia, Vancouver, British Columbia, Canada*

[3] *Department of Economics, School of Economics and Business, University of Sao Paulo, Brazil*

[4] *Center for Food Studies and Research, University of Campinas; Center for Epidemiological Studies in Nutrition and Health, University of Sao Paulo, Brazil*

*Contact information: Tonatiuh Barrientos-Gutiérrez, Center for Population Health Research, National Institute of Public Health, Avenida Universidad 655, Santa María Ahuacatitlán, 62100 Cuernavaca, Morelos, México, [tbarrientos@insp.mx].

Ana Clara Duran, Center for Food Studies and Research, University of Campinas Address: Av. Albert Einstein, 291 Cidade Universitaria, Campinas, SP, 13083-852, Brazil E-mail: anaduran@unicamp.br; Phone: +55 19 3521-2177

Contents

1 Sugar-sweetened beverages selection **4**

2 Adjustment of self-reporting bias on weight and height **4**

3 Model simulations **7**
3.1 Caloric change 7
3.2 Change in body weight 7
3.3 Change in BMI 8
3.4 Change in obesity prevalence 8

4 Reduction in cases with obesity **9**

5 Reduction in healthcare costs **10**
5.1 Obesity health costs averted 11

6 Sensitivity analysis **12**

7 Summary of model structure and parameters **14**

References **15**

Notation

- **POF**, Pesquisa de Orcamentos Familiares - POF) from 2017-2018(POF 2017-2018)
- **IBGE**, Brazilian Institute of Geography and Statistics
- **SSBss**, sugar sweetened beverages.
- **PNS**, Pesquisa Nacional de Saúde (2019)
- k **(superscript)**, kth individual in the sample.
- Δ, change.
- **EI**, energy intake
- BW, body weight function (kg) as a function of time ($BW \equiv BW(t)$).
- **BMI**, body mass index.
- **H**, height.
- t, time in years.
- **pp**, percentage points.
- **US$**, US dollars.
- **R$**, US Reais.

1 Sugar-sweetened beverages selection

Table A shows the categorized beverage items from the Brazilian Household Budget Survey (Pesquisa de Orçamentos Familiares - POF) from 2017-2018 (POF 2017-2018), collected by the Brazilian Institute of Geography and Statistics (IBGE) [1], which were used to estimate the price elasticities of sugar-sweetened beverages (SSBs) by income level.

Table A: Description of the beverage categories selected from the 2017-18 Brazilian Household Budget Survey (Pesquisa de Orçamentos Familiares 2017-18)

**Category**	**Description**
Sugar sweetened beverages	Regular cola and non-cola carbonates, nectars and juice drinks with low content of nectars, packaged ready-to-drink coffee and tea, sports drinks and sugary yogurts.
Alcoholic beverages	Beers, wines, whisky and other alcoholic beverages.
Unsweetened beverages	Bottled water (still and sparkling without sugar), 100% juice (packaged 100% juice obtained from fruits or vegetables by mechanical processes), milk (without sugar) and natural coffee and tea.
Low-calorie or artificially sweetened beverages	Light and diet soft drinks, nectars and juice drinks with low content of nectars, light and diet packaged ready-to-drink coffee and tea, sports drinks and sugary yogurts.

2 Adjustment of self-reporting bias on weight and height

Anthropometric data from POF was self-reported, which could lead to measurement bias in the downward direction. To address this, we adjusted weight and height from POF using measured anthropometric data from the Pesquisa Nacional de Saúde (PNS 2019) using an adjustment method for self-report bias [2]. This method has been previously implemented by Ward et al. [3]. Figure A shows the distribution of self-reported weight and height for males and females in POF compared to measured weight and height in PNS. We observed that females have a higher measurement error than males for both weight and height and it is differently distributed by quantile. In other words, females tend to over-report their height and under-report their weight more than males do. Also, as weight increases, both males and females under-report more their weight.

Fig. A: Comparison between quantiles of self-reported and measured weight and height by sex

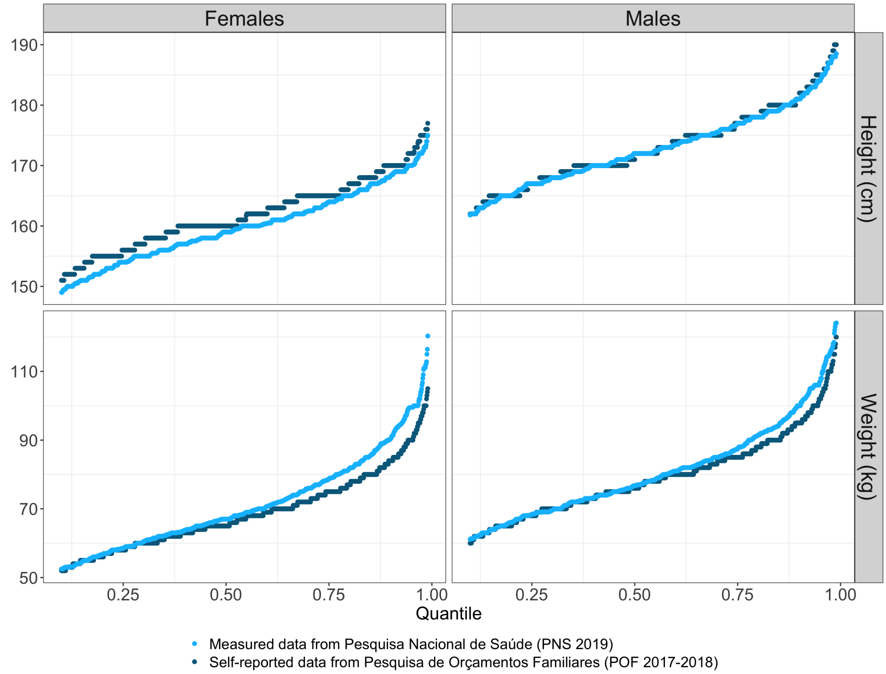

Following the work of Ward et al., we fitted cubic splines to smoothly estimate self-report bias across the weighted quantile distribution of the difference between self-reported and measured weight and height. Then, we calculated the quantile of self-reported weight and height for each respondent in POF and predicted their weight and height difference using the fitted cubic splines. Finally, we added this predicted difference to their self-reported anthropometric data to adjust it. Figure B shows the comparison between self-reported, measured and adjusted anthropometric data.

Fig. B: Comparison between self-reported, measured and adjusted weight and height by sex

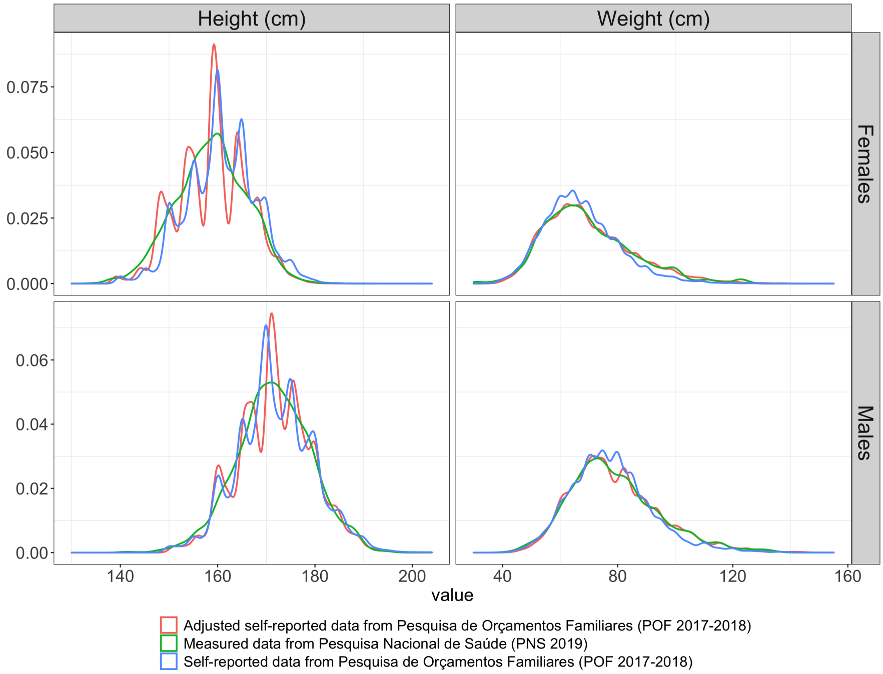

Table B shows the comparison between the observed, self-reported and corrected obesity prevalence. Observed obesity in the Brazilian population was estimated to be 26.8%, higher in females than males, higher in the age group 20-59 years, than 20-39 or 60 years and older and higher in the middle socioeconomic level than low and high socioeconomic level. self-reported data leads to underestimating obesity prevalence in all subgroups. The correction of weight and height we made in this study, leads to obesity prevalence estimations similar to the observed data (the confidence intervals overlap).

Table B: Comparison between self-reported, measured and adjusted weight and height by sex, age group and income status

	Observed PNS 2019		Self-reported POF 2017/2018		Corrected POF 2017/2018	
	Baseline obesity (%)	95% CI	Baseline obesity (%)	95% CI	Baseline obesity (%)	95% CI
Total	26.8	[23.4,30.5]	16.9	[16.3, 17.6]	25.2	[24.4,26.0]
Sex						
female	30.2	[25.8,35.0]	18.0	[17.2, 18.8]	28.8	[27.8,29.8]
male	22.8	[19.7,26.3]	15.7	[14.9, 16.6]	21.2	[20.2,22.2]
Age groups						
20-39	21.0	[16.6,26.3]	14.3	[13.4, 15.2]	21.8	[20.7,23.0]
40-59	34.4	[29.9,39.1]	19.8	[18.7, 20.9]	29.3	[28.1,30.5]
60+	24.8	[20.9,29.1]	17.3	[16.0, 18.5]	24.8	[23.4,26.4]
Income status						
Low	26.0	[21.7,30.8]	15.5	[14.5, 16.4]	22.3	[21.1,23.4]
Middle	28.6	[24.6,32.8]	17.5	[16.5, 18.5]	26.2	[25.0,27.4]
High	26.0	[21.0,31.7]	17.8	[16.5, 19.1]	27.0	[25.5,28.5]

3 Model simulations

3.1 Caloric change

For each individual k in the sample, we calculated the caloric change from sugar-sweetened beverages (SSBs) (ΔEI_k^{SSBs}) and all beverages (ΔEI_k^{Allbev}) after hypothetical tax scenarios as:

$$\Delta EI_k^{SSBs} = -EI_k^{SSBs} \times tax \times elasticity^{SSBs}/10, \quad (1)$$

$$\Delta EI_k^{Allbev} = -EI_k^{bevcat} \times tax \times elasticity^{bevcat}/10, \quad (2)$$

where EI_k^{SSBs} represents the baseline consumption (in calories) from SSBss, $tax = \{2, 3\}$ represents the hypothetical 20 % and 30% tax scenarios, $elasticity^{SSBs}$ corresponds to the price elasticity parameter for SSBss, EI_k^{bevcat} and $elasticity^{bevcat}$ corresponds to the baseline consumption and price elasticity parameters of each beverage category $bevcat = \{$alcohol, other beverages, light or diet beverages$\}$, respectively. Henceforth we will refer to Equations 1 and 2, as ($\Delta EI_k{}^{tax}$) interchangeably.

3.2 Change in body weight

We used a dynamic individual weight change model developed by by Hall et al.. [4, 5, 6] The model considers body weight (BW) as a function of time (t) and depends on individual level characteristics: sex (Sex), initial body weight ($BW(0)$), $height(H(0))$, Age ($A(0)$) and energy intake (EI). The model is implemented in the *bw* package in R [7]. To initialize the model we obtained sex, age, weight and height at t = 0 from the Brazilian Household Budget Survey (Pesquisa de Orçamentos Familiares - POF). Then, considering

changes in caloric intake after hypothetical scenarios of 20% and 30% taxes, we simulated body weight under the tax effect as:

$$BW_k(t) = BW_k^{\text{model}}\big(t + Age_k;\ \text{Sex}_k, \text{Height}_k, \text{BW}_k(0), \Delta EI_k^{tax}\big), \quad (3)$$

where t stands for the number of days after the intervention, k for each individual on the sample, $\text{BW}_k(0)$ the initial body weight and $\Delta EI_k{}^{tax}$, the total caloric changes for each tax scenario.

3.3 Change in BMI

To obtain the expected change in body mass index $BMI_k(t)$ for each individual k, we used Equation (4):

$$BMI_k(t) = BW_k(t)/(H_k)^2, \quad (4)$$

where $BW_k(t)$ represents the estimated individual's body weight (kg) with Hall's model, t stands for the number of days after the intervention, and H_k represents individual's height in meters.

3.4 Change in obesity prevalence

We classified each individual's $BMI_k(t)$ into BMI categories using WHO's cut-off points [8]. We introduced a dummy variable ($BMIcat_k(t)$) defined as:

$$BMIcat_k(t) = \begin{cases} BMIcat_k(t) = 1, & \text{if } BMI_k \geq 30kg/m^2 \\ BMIcat_k(t) = 0, & \text{otherwise,} \end{cases}$$

where $BMIcat_k(t) = 1$ indicates obesity for each individual k in the sample and t stands for the number of days after the intervention.

Then, we calculated the change in obesity prevalence ($\Delta BMIcat_k(t)$) with Equation 5:

$$\Delta BMIcat_k(t) = BMIcat_k(0) - BMIcat_k(t), \quad (5)$$

where k represents each individual in the sample, $BMIcat_k(0)$ corresponds to the baseline BMI category ($t = 0$) and $BMIcat_k(t)$ represents the new BMI category in different time in years.

Finally, since POF is a cross-sectional, multi-stage, probabilistic survey representative of the Brazilian population, we used the R package survey [9] to create summary statistics of $BW_k(t)$, $BMI_k(t)$ and $BMIcat_k(t)$ (each in the overall adult population and in specific subpopulations by sex, SES, and age). For these estimates we accounted for the survey design established as follows:

```
svystr <- svydesign(id = ~id, strata = ~ESTRATO_POF,
weights = ~PESO_FINAL, PSU = ~COD_UPA, data = Adults)
options(survey.lonely.psu = "adjust")
```

4 Reduction in cases with obesity

We estimated the total averted cases of obesity for each year (ΔObesity cases$_{year}$) for the 20% and 30% tax scenarios, considering changes in intake from SSBss and from all berverages, as follows:

$$\Delta \text{Obesity cases}_{year} = \text{Adult population}_{year} \times \Delta \text{Obesity prevalence}_{year}, \quad (6)$$

where Adult population$_{year}$ represents the projected adult population of 20 years or older at $year = 2021, 2022, ..., 2030$ [10]. Table C presents the estimated obesity cases in each year under the counterfactual scenario (no intervention). Tables D and E present the averted obesity cases in each year considering changes in intake from SSBss and all beverages, respectively.

Table C: Estimated population, obesity prevalence and obesity cases in the Brazilian adult population from 2021 to 2030 under no intervention.

Year	Brazilian adult population (20+ years)	Estimated prevalence in 2018	Obesity cases in millions
2021	153,748,413	25.2%	38.7
2022	155,562,137	25.2%	39.2
2023	157,294,648	25.2%	39.6
2024	158,967,408	25.2%	40.1
2025	160,600,523	25.2%	40.5
2026	162,166,361	25.2%	40.9
2027	163,624,456	25.2%	41.2
2028	165,026,861	25.2%	41.6
2029	166,375,797	25.2%	41.9
2030	167,647,336	25.2%	42.2

Table D: Estimated reduction of obesity cases for a 20% and 30% tax considering changes from SSBs.

Year	Absolute reduction (pp)		Cases with obesity (millions)	
	20% tax	30% tax	20% tax	30% tax
2021	-0.94	-1.37	-1.44	-2.11
2022	-1.35	-1.79	-2.10	-2.78
2023	-1.51	-2.00	-2.37	-3.14
2024	-1.58	-2.13	-2.52	-3.38
2025	-1.62	-2.17	-2.61	-3.48
2026	-1.63	-2.18	-2.65	-3.54
2027	-1.63	-2.20	-2.68	-3.60
2028	-1.64	-2.21	-2.71	-3.65
2029	-1.65	-2.21	-2.74	-3.68
2030	-1.65	-2.21	-2.76	-3.71

Table E: Estimated reduction of obesity cases considering a 20% and 30% tax considering changes in all beverages.

Year	Absolute reduction (pp)		Cases with obesity (millions)	
	20% tax	30% tax	20% tax	30% tax
2021	-0.95	-1.38	-1.46	-2.12
2022	-1.36	-1.80	-2.11	-2.80
2023	-1.52	-2.03	-2.40	-3.19
2024	-1.60	-2.14	-2.54	-3.41
2025	-1.65	-2.18	-2.65	-3.51
2026	-1.66	-2.21	-2.68	-3.58
2027	-1.66	-2.23	-2.71	-3.65
2028	-1.67	-2.24	-2.75	-3.70
2029	-1.67	-2.25	-2.78	-3.74
2030	-1.67	-2.25	-2.81	-3.77

5 Reduction in healthcare costs

Obesity costs were obtained from a 2021 international paper estimating overweight and obesity in eight countries, including Brazil [11]. To estimate the cost per obesity case (Table F), we first estimated proportion of costs that were attributable only to obesity, which a review in the US, estimated to be 87%. Obesity costs were estimated to be 33.72 billion USD. We divided by the number of people with obesity and estimated cost per obesity case to be

906.92 USD per year. The cost was translated into 2021 using consumer price index Inflation Rate as 942.5 USD per year.

Table F: Costs of obesity among Brazilian adults in US dollars

	**Direct cost**	**Indirect cost**	**Total cost**
Overweight and obesity cost (billion)	\$16.19	\$22.57	\$38.76
Obesity costs (billion)	\$14.08	\$19.63	\$33.72
Adult population in 2018 (million)		147.5	
Population with obesity (million)		37.2	
Cost per obesity case in 2019	\$378.8	\$528.1	\$906.9
Cost per obesity case in 2021	\$393.7	\$548.8	\$942.5

5.1 Obesity health costs averted

To calculate the reduction in obesity costs over 10 years for 20 and 30% SSBs tax, we used the following equation:

$$\Delta \text{Obesity costs}_{year} = \text{Obesity costs}_{year} \times \text{Obesity prevalence reduction}_{year}, \tag{7}$$

where ΔObesity costs_{year} represents the change in obesity health costs at $year = 2021, 2022, ..., 2030$.

To account for the time lag in morbidity and mortality changes following obesity changes, no cost benefits were estimated during the first three years of intervention. Full benefit of the intervention on direct and indirect costs was modeled in years 2024–2030. Tables G and H present costs averted over the 10 years considering changes in SSBs intake and changes in all beverages after SSB tax.

Table G: Reduction in total (direct and indirect costs) of obesity in Brazilian adults after a 20% and 30 % tax on SSBs (million USD)

Year	Direct costs with 5% discount rate	Indirect costs with 5% discount rate	Direct costs		Indirect costs		Total costs	
			20% tax (millions of dollars)	30% tax (millions of dollars)	20% tax (millions of dollars)	30% tax (millions of dollars)	20% tax (millions of dollars)	30% tax (millions of dollars)
2021	$ 393.7	$ 548.8	0	0	0	0	0	0
2022	$ 374.9	$ 522.7	0	0	0	0	0	0
2023	$ 357.1	$ 497.8	0	0	0	0	0	0
2024	$ 340.0	$ 474.1	-$ 856.5	-$ 1,149.0	-$ 1,194.1	-$ 1,601.9	-$ 2,050.55	-$ 2,750.90
2025	$ 323.9	$ 451.5	-$ 843.9	-$ 1,126.8	-$ 1,176.5	-$ 1,571.0	-$ 2,020.36	-$ 2,697.82
2026	$ 308.4	$ 430.0	-$ 816.2	-$ 1,091.4	-$ 1,137.9	-$ 1,521.6	-$ 1,954.03	-$ 2,612.99
2027	$ 293.7	$ 409.5	-$ 785.8	-$ 1,057.2	-$ 1,095.5	-$ 1,473.9	-$ 1,881.35	-$ 2,531.17
2028	$ 279.8	$ 390.0	-$ 758.0	-$ 1,019.8	-$ 1,056.8	-$ 1,421.7	-$ 1,814.89	-$ 2,441.53
2029	$ 266.4	$ 371.5	-$ 729.9	-$ 981.2	-$ 1,017.5	-$ 1,367.9	-$ 1,747.41	-$ 2,349.12
2030	$ 253.8	$ 353.8	-$ 700.4	-$ 941.6	-$ 976.5	-$ 1,312.7	-$ 1,676.92	-$ 2,254.35
		Total	-$5,491	-$7,367	-$7,655	-$10,271	-$13,146	-$17,638

Table H: Reduction of total (direct and indirect costs) and only direct costs of obesity in Brazilian adults after a 20% and 30 % tax on all beverages (million USD)

Year	Direct costs with 5% discount rate	Indirect costs with 5% discount rate	Direct costs		Indirect costs		Total costs	
			20% tax (millions of dollars)	30% tax (millions of dollars)	20% tax (millions of dollars)	30% tax (millions of dollars)	20% tax (millions of dollars)	30% tax (millions of dollars)
2021	$393.7	$548.8	0	0	0	0	0	0
2022	$374.9	$522.7	0	0	0	0	0	0
2023	$357.1	$497.8	0	0	0	0	0	0
2024	$340.0	$474.1	-$ 864.4	-$ 1,159.3	-$ 1,205.1	-$ 1,616.2	-$ 2,069.47	-$ 2,775.47
2025	$323.9	$451.5	-$ 857.9	-$ 1,136.4	-$ 1,196.1	-$ 1,584.3	-$ 2,053.99	-$ 2,720.65
2026	$308.4	$430.0	-$ 828.0	-$ 1,105.4	-$ 1,154.3	-$ 1,541.1	-$ 1,982.33	-$ 2,646.43
2027	$293.7	$409.5	-$ 797.2	-$ 1,072.4	-$ 1,111.4	-$ 1,495.1	-$ 1,908.55	-$ 2,567.48
2028	$279.8	$390.0	-$ 770.4	-$ 1,035.0	-$ 1,074.1	-$ 1,443.0	-$ 1,844.55	-$ 2,477.97
2029	$266.4	$371.5	-$ 741.8	-$ 995.8	-$ 1,034.1	-$ 1,388.3	-$ 1,775.89	-$ 2,384.10
2030	$253.8	$353.8	-$ 711.8	-$ 955.6	-$ 992.4	-$ 1,332.3	-$ 1,704.25	-$ 2,287.93
		Total	-$5,571	-$7,460	-$7,768	-$10,400	-$13,339	-$17,860

6 Sensitivity analysis

A sensitivity analysis was performed using the observed weight and height from PNS 2019. Because this data is in a different data-set, we used the Hall's model at aggregated level. For that, we estimated the average SSB consumption and BMI for 14 groups: by age (20-39, 40-59, 60+) and socioeconomic status (low, middle, and high) for males and females. We compared results sing the individual and population level model.

Table I shows the comparison between individual and population level data

using adjusted and observed weight and height, respectively. We found small differences in the impact of SSB tax in the Brazilian adult population in both females and males using both methodologies, making our estimations more robust .

Table I: Estimated impact of SSB tax in the Brazilian population stratified by sex and by age and socioeconomic status using the adjusted weight and height (individual model) and the observed weight and height (population model)

	**PNS 2019**		**POF 2017/2018**						
	**Weight kg (measured)**	**Height mt (measured)**	**Age (yrs)**	**Sex**	**Caloric change (kcal (95% CI))**		**Individual model with adjusted weight and height (Change in weight kg (95% CI))**		**Population model with observed weight and height** (Change in weight kg)
Female	69.7	158.7	46.1	Female	-15.3	-15.9,-14.7	-0.75	-0.78,-0.72	-0.78
20-39	68.9	161.6	29.8	Female	-19.6	-20.7,-18.4	-0.91	-0.97,-0.86	-0.93
40-59	73.1	158.5	49.2	Female	-13.3	-14.2,-12.5	-0.68	-0.73,-0.64	-0.71
60+	65.6	154.3	70.2	Female	-10.8	-11.8,-9.8	-0.58	-0.64,-0.52	-0.58
Low	69.0	157.4	45.3	Female	-12.3	-13.4,-11.2	-0.60	-0.65,-0.55	-0.63
Middle	70.2	158.7	46.7	Female	-16.1	-17.1,-15.2	-0.80	-0.85,-0.75	-0.83
High	70.4	161.3	46.2	Female	-17.4	-18.5,-16.2	-0.86	-0.91,-0.80	-0.88
Male	79.0	171.6	44.6	Male	-18.6	-19.5,-17.8	-0.83	-0.87,-0.79	-0.84
20-39	78.7	173.7	29.5	Male	-23.9	-25.3,-22.6	-1.02	-1.08,-0.96	-1.02
40-59	81.5	171.4	49.0	Male	-16.3	-17.5,-15.0	-0.76	-0.83,-0.70	-0.77
60+	75.1	167.8	69.7	Male	-11.4	-12.5,-10.2	-0.53	-0.58,-0.48	-0.54
Low	74.9	169.8	43.5	Male	-14.4	-15.7,-13.2	-0.62	-0.68,-0.56	-0.63
Middle	81.0	172.3	45.1	Male	-19.2	-20.4,-17.9	-0.85	-0.91,-0.79	-0.89
High	82.8	173.5	45.3	Male	-21.8	-23.5,-20.1	-0.99	-1.07,-0.91	-1.02

7 Summary of model structure and parameters

Table J: Model structure, description of parameters and their ranges, primary sources, and important caveats.

Data	Description	Range	Source	Caveats
Model structure				
	Validated dynamic simulation model of adults, that predicts the time course of individual weight change in response to warning labels in Brazil. We chose this model because it considers dynamic physiological adaptations that occur with body weight changes at individual and population level.	Not applicable	Hall et al. 2011	The model assumes a steady state for each individual's body weight, and thus, we could not consider the increase in the obesity trend occurring in Brazil in the last years. - We do not consider potential changes in physical activity over the simulation period and assume a sedentary activity level (1.5). - A main limitation of the model is how total energy intake is estimated. The intervention's impact on body weight relies on the baseline total energy intake estimates via Mifflin-St Jeor's validated formula. This formula has been reported as a low-precision method to estimate total energy intake, yet widely used because other methods are too expensive. This uncertainty increases the variability of weight change results even if the expected caloric change is exactly the same for every Brazilian adult.
Model parameters				
Baseline				
SSB tax intake (kcal)	One 24-hour diet recall	Please see main paper results for more details	POF 2017/2018	Results based on estimated population weights.
Weight(kg)	Self-reported weight from	Mean (95% CI) : 74.3 (74.0, 74.6)	POF 2017/2018	Results based on estimated
	POF 2017/2018 was adjusted fitting a cubic spline with quantiles of measured weight from PNS 2019		and PNS 2019	population weights. Please see the appendix for more details
Height (cm)	Self-reported height from	Mean (95% CI) : 165.0 (164.8, 165.1)	POF 2017/2018	Results based on estimated
	POF 2017/2018 was adjusted fitting a cubic spline with quantiles of measured height from PNS 2019		and PNS 2019	population weights. Please see the appendix for more details
Sex (%)	Individual's reported sex	Females: 52.9 (52.4, 53.4)) Males: 47.1 (46.6, 47.6)	POF 2017/2018	Results based on estimated population weights.
Age (years)	Individual's reported age	Mean (95% CI) : 45.4 (45.1, 45.7)	POF 2017/2018	Results based on estimated
Time	Simulation period	10 years		Results based on estimated population weights.
After intervention				
Change in SSB intake (kcal)	Used our estimated and cross price-elasticities by SES group	Please see main paper results for more details	Purchases data from POF 2017/2018	It is our own estimation, which could be different from others published
Change in weight (kg)	Hall's model at individual level	Please see main paper results for more details	Hall et al. 2011	See caveats above
Costs per obesity person in 2023				
Direct obesity costs	Includes direct medical and non-medical costs. Direct medical costs are estimated as the obesity attributable fraction times total health expenditure.	$451.3		It relies on WHO global health expenditures database and an OECD report for obesity-attributable fractions.
Indirect obesity costs	Includes economic loss from premature mortality, missed days of work (absenteeism) and reduced productivity while at work (presenteeism).	$629.2	Okunogbe 2021	Absenteeism and presenteeism rates associated with obesity are similar to other high-income countries, but variations in labour market behavior in Brazil could change results
Total obesity costs	The sum of direct and indirect obesity costs	$1,080.5		

We included average weight, height, age, obesity prevalence, and proportion of males and females, but our model uses individual parameters from the Brazilian population.

References

[1] Instituto Brasileiro de Geografia e Estatística. Pesquisa de Orçamentos Familiares 2017-2018: antropometria e estado nutricional de crianças, adolescentes e adultos no Brasil. Rio de Janeiro; 2018. .

[2] Instituto Brasileiro de Geografía e Estatística (IBGE). Pesquisa Nacional de Saúde (PNS 2019); 2019. [Online; accessed 01-December-2020]. https://www.ibge.gov.br/estatisticas/sociais/saude/9160-pesquisa-nacional-de-saude.html.

[3] Ward Z, Bleich S, Barret J, Flax C, et al. Projected U.S. State-Level Prevalence of Adult Obesity and Severe Obesity. New England Journal of Medicine. 2019;381(2):2440–2450.

[4] Hall KD, Sacks G, Chandramohan D, Chow CC, Wang YC, Gortmaker SL, et al. Quantification of the effect of energy imbalance on bodyweight. The Lancet. 2011;378(9793):826–837.

[5] Hall KD. Predicting metabolic adaptation, body weight change, and energy intake in humans. American Journal of Physiology-Endocrinology and Metabolism. 2010;298(3):E449–E466.

[6] Hall KD, Jordan PN. Modeling weight-loss maintenance to help prevent body weight regain. The American journal of clinical nutrition. 2008;88(6):1495–1503.

[7] Camacho-García-Formentí D, Zepeda-Tello R. bw: Dynamic Body Weight Models for Children and Adults; 2018. R package version 1.0.0.

[8] World Health Organization. Obesity: preventing and managing the global epidemic. Report of a WHO Consultation.. vol. 894. Geneva: World Health Organization; 2000.

[9] Lumley T. survey: analysis of complex survey samples; 2019. R package version 3.35-1.

[10] Instituto Brasileiro de Geografía e Estatística (IBGE). Projeções da População; 2018. [Online; accessed 01-March-2021]. https://www.ibge.gov.br/estatisticas/sociais/populacao/9109-projecao-da-populacao.html?=&t=outros-links.

[11] Okunogbe A, Nugent R, Spencer G, Ralston J, J W. Economic impacts of overweight and obesity: current and future estimates for eight countries. BMJ Glob Health. 2021;6(10):e006351.
