# Supplementary material for: Estimated reduction in obesity prevalence and costs of a 20% and 30% ad valorem excise tax to sugar-sweetened beverages in Brazil: A modeling study
Source: PLoS Med. 2024 Jul 17;21(7):e1004399. doi: 10.1371/journal.pmed.1004399 (PMC11253955; doi:10.1371/journal.pmed.1004399)
Supplement: Attachment — Submitted filename: Response to reviewers SSB tax Brazil.docx [file pmed.1004399.s002.docx]

Dear Dr. Barrientos-Gutierrez,

Thank you very much for submitting your manuscript "Expected reduction in obesity of a 20% and 30% tax to Sugar-Sweetened-Beverages in Brazil: a modeling study" (PMEDICINE-D-23-02904R1) for consideration at PLOS Medicine.

Your paper was evaluated by a senior editor and discussed among all the editors here. It was also discussed with an academic editor with relevant expertise, and sent to independent reviewers, including a statistical reviewer. The reviews are appended at the bottom of this email and any accompanying reviewer attachments can be seen via the link below:

In light of these reviews, I am pleased to tell you that we would like to consider a revised version that addresses the reviewers' and editors' comments. Obviously we cannot make any decision about publication until we have seen the revised manuscript and your response, and we plan to seek re-review by one or more of the reviewers.

In revising the manuscript for further consideration, your revisions should address the specific points made by each reviewer and the editors. Please also check the guidelines for revised papers at <http://journals.plos.org/plosmedicine/s/revising-your-manuscript> for any that apply to your paper. In your rebuttal letter you should indicate your response to the reviewers' and editors' comments, the changes you have made in the manuscript, and include either an excerpt of the revised text or the location (eg: page and line number) where each change can be found. Please submit a clean version of the paper as the main article file; a version with changes marked should be uploaded as a marked up manuscript.

In addition, we request that you upload any figures associated with your paper as individual TIF or EPS files with 300dpi resolution at resubmission; please read our figure guidelines for more information on our requirements: <http://journals.plos.org/plosmedicine/s/figures>. While revising your submission, please upload your figure files to the PACE digital diagnostic tool, <https://pacev2.apexcovantage.com/>. PACE helps ensure that figures meet PLOS requirements. To use PACE, you must first register as a user. Then, login and navigate to the UPLOAD tab, where you will find detailed instructions on how to use the tool. If you encounter any issues or have any questions when using PACE, please email us at PLOSMedicine@plos.org.

We expect to receive your revised manuscript by January 12 2023 11:59PM. Please email us (plosmedicine@plos.org) if you have any questions or concerns.

***Please note while forming your response, if your article is accepted, you may have the opportunity to make the peer review history publicly available. The record will include editor decision letters (with reviews) and your responses to reviewer comments. If eligible, we will contact you to opt in or out.***

We ask every co-author listed on the manuscript to fill in a contributing author statement, making sure to declare all competing interests. If any of the co-authors have not filled in the statement, we will remind them to do so when the paper is revised. If all statements are not completed in a timely fashion this could hold up the re-review process. If new competing interests are declared later in the revision process, this may also hold up the submission. Should there be a problem getting one of your co-authors to fill in a statement we will be in contact. YOU MUST NOT ADD OR REMOVE AUTHORS UNLESS YOU HAVE ALERTED THE EDITOR HANDLING THE MANUSCRIPT TO THE CHANGE AND THEY SPECIFICALLY HAVE AGREED TO IT. You can see our competing interests policy here: <http://journals.plos.org/plosmedicine/s/competing-interests>.

Please use the following link to submit the revised manuscript:

<https://www.editorialmanager.com/pmedicine/>

Your article can be found in the "Submissions Needing Revision" folder.

To enhance the reproducibility of your results, we recommend that you deposit your laboratory protocols in [protocols.io](http://protocols.io/), where a protocol can be assigned its own identifier (DOI) such that it can be cited independently in the future. Additionally, PLOS ONE offers an option to publish peer-reviewed clinical study protocols. Read more information on sharing protocols at <https://plos.org/protocols?utm_medium=editorial-email&utm_source=authorletters&utm_campaign=protocols>

Please ensure that the paper adheres to the PLOS Data Availability Policy (see <http://journals.plos.org/plosmedicine/s/data-availability>), which requires that all data underlying the study's findings be provided in a repository or as Supporting Information. For data residing with a third party, authors are required to provide instructions with contact information for obtaining the data. PLOS journals do not allow statements supported by "data not shown" or "unpublished results." For such statements, authors must provide supporting data or cite public sources that include it.

We look forward to receiving your revised manuscript.

Best wishes,

Philippa Dodd - PLOS Medicine, on behalf of:

Katrien Janin, PhD

PLOS Medicine

[plosmedicine.org](http://plosmedicine.org/)

-----------------------------------------------------------

COMMENTS FROM THE ACADEMIC EDITOR

I'm supportive of the paper, but have a few comments.

1. I don't see any particular rationale for essentially replicating similar studies that have been conducted elsewhere beyond no-one's done it in Brazil yet. It would be helpful to understand whether the authors expect price elasticity of SSBs in Brazil to be different from elsewhere and why. I guess really my underlying question is why are modelling studies from elsewhere not generalisable to Brazil? I think there are probably some good reasons, but it would be good to be explicit.

Thank you for this relevant comment. There are many variables involved in the estimation of potential impacts of taxes to SSB. In Latin America few studies have analyzed the potential impact of SSB taxes beyond Mexico, Chile and Argentina. Brazil is the largest country in the region, yet, there is no study assessing potential impacts. Beyond the relevance of Brazil, there are several variables that preclude the generalization of potential impacts across countries:

1. Elasticity: this is highly variable across countries. Meta-analytical estimates found 15.9% reduction as the mean estimate across 33 studies, yet the confidence interval ranged from 10.8% to 21.1%, which is a very wide set of potential effects. Each country has a specific elasticity, depending on the product cost and disposable income, so estimating local data is very relevant.
2. Baseline SSB consumption. Country-level consumption is widely variable for SSB, but particularly for SSB that are amenable to taxation (industrialized). While some countries consume large volumes of homemade SSB and little industrialized SSB, for others it is the opposite. The only way to model the potential impact of a tax is to estimate local consumption patterns for both taxed and untaxed SSB.
3. Body weight. Body weight is our primary outcome, as we are using those results to estimate health and economic benefits. Body weight is also very variable across countries, even within the Latin American region. As an example, while Brazil has a 20% obesity prevalence, in Mexico the prevalence is close to 36%, which makes the potential impact of any obesity-related policy intervention different.
4. Costs. The cost structure of obesity is also widely different across countries. For instance, costs in the US are not the same as Brazil, thus, we cannot expect for the same policy to have the same impact between these countries.

These four variables are the most relevant to our estimation procedure, and as you can see there is no way to directly predict the impact of taxation on consumption, body weight or health costs without proper modeling estimation. Finally, Brazil is about to implement a 20% tax on SSBs; having Brazilian data on the expected impact of the tax could be helpful for policy discussion.

We added this to the authors summary, as follows:

- *In May 2022 in Brazil, a bill was approved at the federal senate to apply a 20% tax on SSB.*
- *Estimating the potential impact of an SSB tax on obesity and cost savings could help inform policy decision making in the country and in the region.*

2. I don't understand how NOVA would be used to classify beverages into the four categories listed - NOVA categorises based on degree of industrial processing.

We classified the beverages into the four listed categories because both the nutritional composition and the drinking behavior of those four groups are markedly different. According to NOVA, food and beverages with additives of 'cosmetic use', which include non-sugar sweeteners, flavors, and colors, are classified as ultra-processed foods and beverages. We rephrased the sentence below for more clarity , which was added to the methods sections as follows.

*“We divided beverages into four categories: (1) sugar-sweetened beverages, (2) alcoholic beverages, (3) unsweetened beverages, and (4) low-calorie or artificially sweetened beverages (light/diet). Table A in S1 Appendix describes the beverage items included in each category.”*

3. Consumption was based on individual data for participants aged 20y+. In many countries SSB consumption peaks in late childhood. So I expect that their estimates of population consumption are underestimates. This could be considered further.

Thanks for the comment. Our study focuses exclusively on adults, thus, we did not assess potential impacts on children. To make this clear we added the following to the limitations section:

*“Finally, we simulated this intervention in the adult population, so further reductions in obesity prevalence are to be expected in children and adolescents, that may change dietary habits and reduce weight as they transition into adulthood.”*

COMMENTS FROM THE EDITORS

GENERAL:

1. For in-text reference, citations are placed within square parentheses and should precede punctuation. (e.g see line 83: ‘… metabolic syndrome, and cancer.[1–3] Governments … ‘ should be changed to ‘ ‘…metabolic syndrome, and cancer [1–3].’‘

Please amend throughout.

Thank you for pointing this out. We changed it throughout the text.

2. Where you provide CI values, please also provide p values for all results where appropriate (e.g see Table 2 and 3), check and amend throughout. For p values, please report as p<0.001 and where higher as 'p=0.002'. Please add the statistical method used to your method section.

We are not doing a formal hypothesis test as the values were estimated to be used as inputs for the weight change model. For table 2, to estimate changes in calories we multiplied the price-elasticity by SES group by the baseline caloric consumption at individual level. Then, we grouped it by different socioeconomic status and generated confidence intervals based on the uncertainty of the national health survey. To acknowledge the uncertainty, we added a table footnote:

** Confidence intervals were generated based on the uncertainty of POF 2017-2018.*

For Table 3, weight change is estimated through microsimulation using Hall's model. This is a deterministic model, in which no randomness is involved in the estimation, giving always the same output (one weight change solution for each individual). The CIs only consider the sources of error captured by the survey data. To acknowledge this we also added a table’s note, as follows:

** Confidence intervals were generated based on the uncertainty of POF 2017-2018.*

3. We also invite you to report p values to consistently to the third decimal digit - thousandths (e.g see table Table 1).

We have changed the Table 1 as follows:

| Table 1: Price elasticities of SSB by income tertiles, 2018. | | | | | |
| --- | --- | --- | --- | --- | --- |
|  |  | Sugar sweetened beverages | Unsweetened beverages | Alcohol beverages | Light/diet beverages |
| Dependent variable: Log of Quantity Acquired (in Liters) | | | | |  |
| Low income | *Elast.* | -1.241*** | 0.046*** | -0.122** | -0.174 |
|  | *s.e.* | -0.015 | -0.017 | -0.053 | -0.124 |
|  | *p-value* | <0.001 | 0.006 | 0.022 | 0.160 |
| Middle income | *Elast.* | -1.186*** | 0.030 | -0.076 | 0.094 |
|  | *s.e.* | -0.019 | -0.021 | -0.047 | -0.11 |
|  | *p-value* | <0.001 | 0.150 | 0.108 | 0.397 |
| High income | *Elast.* | -1.126*** | 0.066*** | -0.141*** | 0.201** |
|  | *s.e.* | -0.022 | -0.025 | -0.048 | -0.084 |
|  | *p-value* | <0.001 | 0.007 | 0.003 | 0.018 |
| *Observations (n)* | | 18,876 | 27,488 | 4,263 | 383 |
| Notes: All regressions used microdata from POF 2017-2018. Income levels were divided using the tertiles of the distribution of the income per capita of the households). Other controls include logarithm of prices of other products (11 categories included), of income (in levels and squared), characteristics of household head (schooling, age, gender, race), total number of household members and total number of children living within the household (all ages). All regressions are weighted using sample survey weights. Standard deviations are clustered by socioeconomic strata. P-values: * 0.10 ** 0.05 *** 0.01 | | | | | |

MODELLING STUDIES

Of all authors who submit a modelling study we ask for inclusion of specific items, derived from Geoffrey P Garnett, Simon Cousens, Timothy B Hallett, Richard Steketee, Neff Walker. Mathematical models in the evaluation of health programmes. (2011) Lancet DOI:10.1016/S0140-6736(10)61505-X.

Please ensure all the items listed below are included with your manuscript. Please review the list below and confirm/revise as necessary:

i) Please provide a diagram that shows the model structure, including how the disease natural history is represented, the process and determinants of disease acquisition, and how the putative intervention could affect the system.

Included in the main paper in figure 1.

ii) Please provide a complete list of model parameters, including clear and precise descriptions of each parameter, together with the values or ranges for each, with justification or the primary source cited, and important caveats about the use of these values noted.

We added a complete list of the model parameters in appendix, as requested:

iii) Please provide a clear statement about how the model was fitted to the data [including goodness-of-fit measure, the numerical algorithm used, which parameter varied, constraints imposed on parameter values, and starting conditions].

We changed the description of the model to include the requirements, as follows:

*To simulate weight changes attributable to the SSB tax on the Brazilian population, we used a microsimulation approach, a dynamic model developed by Hall et al [22]. The model considers the dynamic physiological adaptations to altered body weight that lead to changes of the resting metabolic rate and the energy cost of physical activity [22]. It has been previously implemented to estimate the impact of the sugar-sweetened beverages tax [9,23,24]. The model considers changes in extracellular fluid, glycogen, and fat and lean tissues caused by the change in caloric consumption but keeping the physical activity constant at the individual level. Bodyweight is the result of the sum of fat mass and fat-free mass, and it is determined by the model using a system of ordinary differential equations. To initialize the model and obtain resting metabolic rate, we used sex, age, weight and height from POF 2017-2018 assuming all adults were sedentary (physical activity level=1.5). The model is implemented in the bw package in R (*[*https://rdrr.io/github/INSP-RH/bw/*](https://rdrr.io/github/INSP-RH/bw/)*). Equations and model implementation can be found elsewhere [22], table XX presents the model parameters and section 3 in S1 Appendix presents an overview of the simulation model.*

iv) For uncertainty analyses, please state the sources of uncertainties quantified and not quantified [can include parameter, data, and model structure].

We added this in the methods sections as follows:

*Uncertainty*

*Obesity prevalence considered the uncertainty of POF 2017-2018*

v) Please provide sensitivity analyses to identify which parameter values are most important in the model. Uncertainty estimates seek to derive a range of credible results on the basis of an exploration of the range of reasonable parameter values. The choice of method should be presented and justified.

We had two different sources of information for the body weight input, one aggregate measured and individual adjusted self-reported, so we ran two different simulations to compare the individual level model (main analysis) and aggregated-level model (sensitivity analysis). The information of sensitivity analysis was already included in the manuscript.

vi) Please discuss the scientific rationale for this choice of model structure and identify points where this choice could influence conclusions drawn. Please also describe the strength of the scientific basis underlying the key model assumptions.

Hall’s weight change model uses a dynamic mathematical modeling approach to human metabolism that integrates knowledge about how the human body responds to changes in diet and physical activity, which other models fail to consider. Additionally, It provides estimates at an individual level, which allows estimating stratum-specific effects. Table XX in appendix discusses the model structure and its caveats.

DATA AVAILABILITY:

Thank you for making all the data fully available without restriction. Given that your study is a modelling study, you are also responsible for providing the source code needed to replicate the study's findings in a repository (such as GitHub, SourceForge or Bitbucket) or a cloud computing service (such as Code Ocean). Please explain in the Data Availability Statement how readers can access or will be able to access the shared code.

We added a repository in Github with the database and added the following text to the methods section.:

The script and database for this analysis can be found in the following repository:

https://github.com/INSP-RH/Brazil_SSB_Tax

ABSTRACT:

In the last sentence of the Abstract Methods and Findings section, please describe the main limitation(s) of the study's methodology.

We added the following in the abstract:

*Study limitations include using a quantile distribution method to adjust self-reported baseline weight and height, which could be insufficient to correct for bias; also, weight, height and physical activity were assumed to be steady state under no intervention.*

AUTHORS SUMMARY:

At this stage, we ask that you include a short, non-technical Author Summary of your research to make findings accessible to a wide audience that includes both scientists and non-scientists. The Author Summary should immediately follow the Abstract in your revised manuscript. This text is subject to editorial change and should be distinct from the scientific abstract. Please see our author guidelines for more information: <https://journals.plos.org/plosmedicine/s/revising-your-manuscript#loc-author-summary>

Ideally each sub-heading should contain 2-3 single sentence, concise bullet points containing the most salient points from your study.

Why Was This Study Done?

- *The consumption of sugar-sweetened beverages (SSB) is associated with obesity, metabolic diseases, and incremental healthcare costs.*
- *In May 2022 a bill was approved at the federal senate in Brazil, to introduce a 20% tax on SSB.*
- *Estimating the potential impact of an SSB tax on obesity and cost savings could help inform policy decision making in the country and the region.*

What Did the Researchers Do and Find?

- *We estimated own and cross price elasticities by socioeconomic status of introducing a 20% tax to SSB and estimated expected changes in consumption. If a 20% tax is introduced we would expect a 16.9 kcal/day/person reduction among Brazilian adults.*
- *We used an individual dynamic weight change model to translate consumption reductions in reductions in obesity. We estimated an expected 6.3% reduction in obesity, which should lead to* USD 22.3 billion *healthcare cost savings over 10 years.*
- *Larger benefits of the tax are expected to be experienced by younger adults and by people in the high-income tertile.*

What Do These Findings Mean?

- *The implementation of the SSB tax is expected to produce important population weight and obesity reductions, while saving health-related costs.*
- *Main limitations of our analysis include the use of a 24-hour nutrition recall questionnaire for SSB consumption, which tends to underestimate consumption, and the assumption of no change in weight under the no-intervention scenario.*

In the final bullet point of ‘What Do These Findings Mean?’ Please include the main limitations of the study in non-technical language.

In the last bullet we added the main limitation, as seen in our prior answer.

DISCUSSION:

In line 338: Please avoid assertions of primacy. We suggest you add ‘ to our knowledge’ or something alike.

We changed the overall sentence as follows:

*While the SSB tax is being discussed in Brazil, scarce information exists about the potential impact of a 20 and 30% SSB tax on obesity.*

In line 398 the term "trend" is used. The term trend should be used only when the test for trend has been conducted. Please revise accordingly.

Thank you, we were not referring to a statistical test-for-trend. We changed the limitation to make it clearer, as follows:

*Our estimates depend on a steady-state assumption for body weight, implying a constant obesity prevalence over time; however, the prevalence of obesity in Brazil has increased over time (11.8% to 20.3% between 2009 and 2019). The impact of this assumption depends on the drivers of the obesity trend in Brazil, with the worst-case scenario being an overestimation of the effect that could occur if obesity increases are driven by products not included in the SSB tax. However, SSB are recognized as important drivers of the obesity epidemic in the world. Despite this, future studies should aim at including obesity trends into models.*

REFERENCES:

As mentioned above, for in-text reference, citations are placed within square parentheses and should precede punctuation. Please amend throughout.

Done

For online references (e.g see reference 1), please use the following format: [accessed on 2 Apr 2021] instead of [cited 2 Apr 2021].

Done

FIGURES and TABLES:

Please ensure you re-define abbreviations in the legends of your figures and tables (e.g Figure 1, Table 1, 2 etc.).

Done

COMMENTS FROM THE REVIEWERS:

Reviewer #1: Dear authors, this is an important study, which aimed to estimate the price elasticity of sweetened drinks and other drinks and evaluate the impact of taxation on sweetened drinks on the prevalence of obesity in Brazil. There are few studies that have evaluated the impact of fiscal measures to reduce SSB consumption on health outcomes, especially in low- and middle-income countries. The study makes important contributions to the scientific community in the area of impact assessment of tax policies.

1- Title: I suggest that the aspect of impact on cost be incorporated into the title as this was an important objective of the study.

Expected reduction in obesity *prevalence and obesity costs* o*f a 20% and 30% ad valorem excise tax to Sugar-Sweetened Beverages in Brazil: a modeling study*

2. Page 3 line 86: I suggest removing the word recent, as this is a study published in 2019.

*We removed it.*

Introduction:

3. In 2022, Brazil approved a bill that is still under analysis by the economic affairs committee that provides for a 20% tax on sugary drinks. I think it is important to include this information in the introduction to inform the reader where the country is in this fiscal policy.

The authors should make it clearer in the introduction about the importance of the analysis carried out in this study, that is, why it is relevant to estimate the potential impact of SSB taxation on both obesity and costs in the health system.

We added this sentence in methods to add the background of a 20% tax being discussed in the Senate, as follows:

*In 2022, the Senate committee approved a bill that is now under analysis by the economic affairs committee to impose a 20% tax on SSBs*.

4. Other studies in this regard have already been published in Brazil. It is important to include. Enes, C.C.; Rinaldi, A.E.M.; Nucci, L.B.; Itria, A. The Potential Impact of Different Taxation Scenarios towards Sugar-Sweetened Beverages on Overweight and Obesity in Brazil: A Modeling Study. Nutrients 2022, 14, 5163. https:// [doi.org/10.3390/nu14235163](http://doi.org/10.3390/nu14235163)

Thanks for pointing out this study. We discussed this in your question number 8.

Methods

5. Why were weight and height data not used directly from PNS 2019, since POF 2017-2018, from which consumption data were obtained, was carried out in a period close to PNS 2019? Although it is not possible to combine the databases at the individual level, when establishing the study population >= 20 years old, it is possible to cross-reference consumption data with obesity prevalence data, as the research was carried out at similar times. This way there would be no need to calculate adjustments for weight and height.

As we used a microsimulation approach, we needed data at individual level from both SSB consumption and weight and height. Since datasets can not be combined at the individual level, we had to rely on self-reported height and weight. However, we did a sensitivity analysis using group averages of measured weight and height by 14 groups by age, SES and sex. We implemented Hall’s dynamic model using the averages, generating the expected weight reduction in kilograms for each group. We changed the subtitle in the methods section to make it clearer, as follows:

*Sensitivity analysis: measured weight and height from PNS 2019*

6. The dynamic model proposed by Hall et al predicts that 50% of the expected weight loss will occur in the first year after taxation and 95% of the reduction by the end of the third year of taxation. How was this considered in the model to calculate weight reduction over a 10-year period?

We used a microsimulation weight change model that considers the dynamic physiological adaptations to altered body weight that lead to changes of the resting metabolic rate and the energy cost of physical activity. Thus the model considers the adaptation of body weight. We simulated for a 10-year period to make sure 100% of the effect was captured. More information on Hall's model was provided to improve clarity of the model, both in the main paper (section reduction in body weight, BMI and obesity prevalence) and appendix (Section 7 with the model structure and parameters).

Results

7. Page 7 line 272: remove the indication of % -25.3% as it is kcal

Thanks, we removed it

Discussion

8. In line 338, the authors state that this is the first study in Brazil that estimated the impact of SSB taxation on body weight. There is a mistake in this statement because a study was published in 2022 that also proposed to evaluate the impact of SSB taxation. Enes, C.C.; Rinaldi, A.E.M.; Nucci, L.B.; Itria, A. The Potential Impact of Different Taxation Scenarios towards Sugar-Sweetened Beverages on Overweight and Obesity in Brazil: A Modeling Study. Nutrients 2022, 14, 5163. https:// [doi.org/10.3390/nu14235163](http://doi.org/10.3390/nu14235163)

Even the results found in this study were different from those presented in this article and the possible reasons for these differences should be discussed and explored

Thanks. We removed that sentence and added a paragraph comparing results, as follows:

*While the SSB tax is being discussed in Brazil, scarce information exists about the potential impact of a 20 and 30% SSB tax on obesity.* Based on our modeling exercise, we estimated that a 20% tax could result in a 6.7% reduction in obesity, increasing to 9.1% under a 30% tax. *This estimate is higher than what was previously estimated in Brazil, in which a 20% tax was predicted to increase obesity by 1.3 % in men and by 2.4% in women. Differences in estimates are likely due to discrepancies in the method of estimation of own- and cross-price elasticities, as well as in the caloric densities used. In our study we predicted a 17.3 kcal/day/person reduction, considering own and cross-price elasticities, yet, the study by Enes, et al., (2022), predicted an increase of 8 kcal/day/person, also considering own and cross price elasticities. Both estimates were generated using the same dataset (POF, 2017-2018), yet, Enes, et al., estimated used censored equations at the household level, while we estimated elasticities at the sample unit level, to avoid censored data. Differences in the categorization of food and beverage items and in the caloric content of products could have made a difference, too. Given the observed discrepancy we searched for independent assessments of elasticities in Brazil, and found a recent report by Venson, et al., using the same data - Brazilian Household Budget Survey (POF) 2017-2018 - with a QUAIDS model together with censored demand and household data. Using the elasticities by Venson, we found similar results to our analysis, suggesting that this more sophisticated approach concurs with our analysis. It is important to highlight that our approach allowed us to estimate elasticities and effects by income tertile, an important condition to assess the expected impacts by socioeconomic status.*

We separated this from the paragraph discussing equity, as follows:

We observed important differences in the expected obesity impact by income tertiles. High-income groups are expected to reduce their obesity prevalence by 7.8%, compared to 5.4% in low-income groups. This could be explained by differences in baseline consumption of SSB, which were 86.7 kcal/day for high-, 74.0 kcal/day for middle-, and 53.5 kcal/day for low-income groups. Also, baseline obesity prevalence was 27.0% in high, 26.2% in middle, and 22.3% in low-income groups. This, along with greater baseline SSB consumption, explain the larger expected reductions in BMI among high-income individuals. We found that a 20 and 30% excise tax on SSB will produce benefits for all income groups as it will reduce obesity levels in all SES groups, however investment of tax revenue could further benefit lower-income groups if used to fund redistributive social policies [32].

Reviewer #2: This is a well-written paper on a topic that is of interest to a wide health readership. The methods are sound and the findings are novel. The conclusions are supported by the results. The results are immediately relevant to Brazil, and also of value to similar countries in the region and around the world.

The derivation of price elasticities, by income tertile, using Brazilian data, is a clear strength of this work. So are the use of a microsimulation model with Hall et al's energy balance, and the correction applied to self-reported height and weight, the latter with an alternative approach in the sensitivity analysis for validation purposes.

I have a few comments, but none detract from the great work you have done for this paper.

The approach to estimating health care cost is suitable for ballpark estimates but is likely to underestimate the true health care cost savings by a considerable margin, as the authors duly indicate in their discussion. Not mentioned is that the cost per case is the same across ages, whereas in reality it would rise with age as the prevalence of obesity-related conditions rises. Given population aging, not factoring this in would lead for further underestimation of the cost savings.

Thanks for this comment. The available literature about obesity costs in Brazil is very heterogeneous, ranging from USD 133.8 million to USD 6.3 billion per year (47 times the lowest estimation). We were using the obesity cost as US$ 365.7 million from Nilson EAF in 2018. When divided by people with obesity, it gave us US $10.34/person/year, which we agree is an underestimation. Yet, after reading your comment we looked for updated estimates and found a better reference; thus, we re-estimated the cost savings of the SSB tax. The new cost is closer to what has been estimated in other countries and includes both direct and indirect costs. In the new estimation, instead of using the direct cost of US $10.34/person/year, we are using US$451.3/person/year for direct costs and US$629.2/person/year for indirect costs, which together sums US$1080.5 /person/year.

We changed methods, results and discussion to include the new estimation. We also included the limitation of using the same obesity cost by age. As we are modeling over 10 years and the population is aging, it is reasonable to expect that the proportion of people with obesity will be higher, which should lead to higher "average" costs.

Methods

*Obesity costs were obtained from a 2021 publication which estimated direct and indirect costs attributable to overweight and obesity for eight countries, including Brazil [26]. The total cost of obesity in Brazil was US$ 38.76 billion (16.19 direct and 22.57 indirect costs), using a societal perspective. Direct costs were estimated as the obesity-attributable fraction of healthcare expenditures and indirect costs included economic loss from premature mortality, missed days of work (absenteeism) and reduced productivity while at work (presenteeism) [26]. Using a systematic review that estimated that 87% of the overweight and obesity costs were attributed to obesity, we estimated the obesity costs in Brazil to be US$33.72 billion. In 2018, we estimated a 25.2% obesity prevalence in the adult population in Brazil using POF, resulting in 37.2 million persons with obesity. Dividing the annual cost of obesity by the total number of obesity cases, we estimated an annual cost per capita of US$ 906.92. Using the Consumer Price Index Inflation rate, we converted costs from 2019 to costs in 2023 and obtained an annual cost of US$ 1085.5. Finally, we multiplied the absolute yearly reduction in obesity prevalence by the obesity costs to estimate the total obesity cost savings.*

*Results*

*Figure 2 shows the expected obesity reduction in cases and potential cost savings 10 years after implementing the SSB tax, using own-price elasticities. A 20% tax is expected to reduce the number of people living with obesity by 2.8 million and by 3.7 million with a 30% tax; this would translate into direct cost savings of $US 22.3 billion with a 20% tax and $US 30.0 billion with a 30% tax. Using cross-price elasticities, these estimates increase, so that the reduction in the number of people living in obesity reaches -2.8 million and -3.8 million for a 30% tax; cost savings increase to $US 22.6 billion with a 20%, and $US 30.3 billion with a 30% tax.*

*Discussion*

A SSB tax in Brazil is expected to produce important savings related to obesity prevention. We estimated a reduction of US$ 22.6 billion in obesity costs ten years after the implementation of a 20% SSB tax, US$ 9.4 billion including only direct costs. This 9.4 billion reduction is smaller than what has been estimated in the US (US$ 23.6 billion) but higher than for Mexico (US$ 159.5 million) for a similar tax increase (20%) and a similar period (ten years).[12,33] We compared our direct costs, because in both studies only direct costs were included. Differences could be due to different costs per obesity case, which vary by health system, but also to the number of people with obesity that could be averted (differences in obesity prevalence). Finally, our cost-analysis does not consider the potential benefits derived from the social investment of the tax revenue, particularly if revenue is directed at strengthening obesity prevention measures, which can further increase the health benefits from the tax.

Because the available literature in obesity costs in Brazil is very heterogeneous, our findings on cost reduction also have limitations. A systematic review found that obesity costs in Brazil ranged from USD 133.8 million to USD 6.3 billion per year, but only one study included direct and indirect costs [35]. We relied on an international estimation that includes 8 countries, uses a societal perspective, and includes both direct and indirect costs. Since we are using average obesity costs we are not considering that over the 10 years simulated, the Brazilian population will age, which could affect the average cost of obesity; thus, our cost per person could be underestimating the cost at the end of the simulation period.

Line 249 / 394 / S1 appendix section 5: I believe that usually, the health system perspective does include out-of-pocket expenses. The perspective used is perhaps better described as a 'payer perspective', as per an ISPOR Special Task Force Report (Garrison et al, ViH 2018; <https://doi.org/10.1016/j.jval.2017.12.006>) (although in that guidance the term 'health care sector perspective' is used).

Thank you for your comment. You are right. We changed the cost savings estimation and now it considers a societal perspective, as follows:

*The total cost of obesity in Brazil was estimated to be US$ 38.76 billion (16.19 direct and 22.57 indirect costs), using a societal perspective. Direct costs were estimated as the obesity-attributable fraction of healthcare expenditures and indirect costs included economic loss from premature mortality, missed days of work (absenteeism) and reduced productivity while at work (presenteeism) [26].*

Consider reporting on the results of sensitivity analysis in results section, even if it's in one sentence.

We added the sensitivity analysis in the results section as follows:

*The caloric change was translated into -0.75 kg in females and -0.83 kg in males. In our sensitivity analysis using measured weight and height and hall´s model at aggregated level, females were estimated to reduce 0.78 kg and males 0.84 kg (Table H in appendix).*

Line 411: Add a reference for the statement that "In 2017, the World Health Organization recommended that all countries should implement an SSB tax of at least 20%."

We added the reference as follows:

World Health Organization. Fiscal policies for diet and prevention of noncommunicable diseases: technical meeting report, 5–6 May 2015, Geneva, Switzerland. Geneva: World Health Organization; 2016. <https://www.who.int/publications/i/item/9789241511247>

S1, Fig. B: The adjustments made to height seem to have exacerbated the clustering of values around round numbers, no doubt due to the splines. If there were a way to smooth the adjusted curves, that would lead to more plausible (and elegant) distributions. However, I do not expect this would have much impact on the results or bias them.

Thank you for your comment. Since the adjustment for self-report bias is a complex problem to solve, we decided to use a previously implemented method by Ward et al. We acknowledge that there is still room for improvement, however, in our sensitivity analyses differences between self-reported and measured anthropometric indices were very small (See Table B in Appendix), which suggests that despite limitations the correction works. As you mention, smoothing those values will not affect average estimates.

S1, Table D: typo "absolut"

Changed, thanks!

S1, Table G: indicate units used

We added the units, in billion USD.

Reviewer #3: Thank you for the opportunity to review this article submitted to PLOS Medicine, titled "Expected reduction in obesity of a 20% and 30% tax to Sugar-Sweetened-Beverages in Brazil: a modeling study" by Basto-Abreu and colleagues. Authors provide critically important analyses of the price elasticity of demand of sugar sweetened beverages (SSBs) estimated from nationally representative data sources, as well as projections of the impact of a 20% and 30% price increase on consumption, obesity prevalence, and health care spending. Given that SSB taxes and other SSB reduction policies are becoming increasingly popular globally, this work will be incredibly useful for policymakers who might be considering legislation aimed at restoring the higher tax rate on SSBs in Brazil. The manuscript is clear and well-written, and the analyses are appropriate and described in adequate detail. Overall, I do think the authors need to qualify their simulation results of the impact of a tax on obesity and health care spending a bit more given the limitations of their approach. Lack of high quality data otherwise may preclude more complicated and nuanced analyses, so this analysis is still very useful and informative, but I would have liked to see more detailed comparisons to other published cost-effectiveness analyses that have been done for national SSB taxes. I have several additional minor suggestions for revision, detailed below.

Minor comments

* Recommend specifying the type of tax being modeled in the title: "20% and 30% tax to Sugar-Sweetened Beverages" could be rephrased as "20% and 30% ad valorem excise tax to Sugar-Sweetened Beverages" for clarity, to distinguish this from a tax that increases the price of SSBs based on the volume of the product.

We changed the title as follows:

Expected reduction in obesity *prevalence and obesity costs* o*f a 20% and 30% ad valorem excise tax to Sugar-Sweetened Beverages in Brazil: a modeling study*

Methods

* Recommend rephrasing "low-calorie sweetened beverages" to "low-calorie or artificially sweetened beverages" given the information in Table A in S1.

Thanks, we changed the name of the category to “low-calorie or artificially sweetened beverages"

* Line 157-159: What is "neighboring region", and why weren't households that reported zero consumption just excluded from the elasticity analysis? How would they contribute to the relationship between quantity demanded and price?

The economic theory posits that zero consumption represents a choice made by consumers who assess prices and opt not to make a purchase. However, a challenge arises in that we only have price data for consumers who have actually bought the product. To address this issue, empirical studies often substitute the unobserved price with prices from neighboring regions, assuming that these neighboring areas generally experience similar market prices. In our context, "neighbor" refers to the geographic unit within the sample. In the IBGE sampling methodology, we observe a specific number (stratum) corresponding to families located in close geographic proximity. Therefore, we have utilized this definition to assign prices to consumers who did not make purchases during the week of data collection. We have included a clarification in the revised manuscript.

*The economic theory posits that zero consumption represents a choice made by consumers who assess prices and opt not to make a purchase. If a household reported zero consumption of an item, we approximated its price by the median of the neighboring regions, under the assumption that those areas generally experience similar market prices.*

* Line 167: What levels of income were included in the model as covariates?

We added income and squared income as covariates and “income tertiles” interacted with prices. We have made it clearer in the revised manuscript.

* Line 173: For the interaction model -- were the main effects of log price of each food also included in this model? Or does this model only include the interactions between each log price and tertiles of income? If so, authors should clarify the implication of this modeling assumption for the interpretation of their results.

Yes, all the prices (SSBs, unsweetened Beverages, alcoholic Beverages, snacks, sugar, other processed foods, in-natura meats, in-natura foods, fruits and vegetables, low-calorie or artificially sweetened beverages) were interacted with income tertiles.

* Line 178: What is the size of the subsample of participants with two-day 24h recalls? As a sensitivity analysis, it would be useful to compare intakes in this subsample by day just to ensure that the one-day 24h recall is valid.

The subsample of participants with two-day 24h food recalls is 31,762, which expands to 120.7 millions. Because of our choice to estimate the energy intake of the entire Brazilian adult population (n=37,689 expanded to 147,8 millions), and so we could compare our findings to previous studies that did not have repeated 24-h food recalls, we decided to use estimates from the single 24h food recall. We estimated the consumption using the two-days and the average consumption was 82.5 kcal (80.5, 84.5) in comparison to 71.8 kcal (69.2, 74.4) using a single day and we are aware we may be underestimating the overall population energy intake. We changed the methods sections as follows:

*Food consumption was assessed using a single 24h recall because two-day information was collected in a smaller subsample (*31,762) *and sampling weights did not expand to the overall Brazilian population.*

* Line 214: Since 'bw' does not appear to be a standard package on the CRAN repository, please provide a link to the code base to aid replication.

Thanks. We added the link in the methods section as follows:

The model is implemented in the bw package in R (<https://rdrr.io/github/INSP-RH/bw/>)

* Health care costs across countries are of course quite different, but studies of the direct cost of obesity from US settings have estimated the per capita excess spending due to obesity as much higher (Cawley et al. 2021 report an estimate of $2,505 per year per person)

Cawley, John, et al. "Direct Medical Costs of Obesity in the United States and the Most Populous States." Journal of Managed Care & Specialty Pharmacy, vol. 27, no. 3, Mar. 2021, pp. 354-66. PubMed, <https://doi.org/10.18553/jmcp.2021.20410>.

Could the authors explain the discrepancy between these estimates and the number used in this analysis of US $10.34 per person per year? The Nilson et al. study cited by the authors appears to separate out the cost of obesity from that of hypertension and diabetes, though these are clearly connected. It would help if additional details on what is included in the authors' costing of obesity are provided, either here in the Methods or in the Discussion as a potential springboard for comparisons to other studies.

Thanks for this comment. The available literature about obesity costs in Brazil is very heterogeneous, ranging from USD 133.8 million to USD 6.3 billion per year (47 times the lowest estimation). We were using the obesity cost as US$ 365.7 million from Nilson EAF in 2018, but when divided by people with obesity, it gave us US $10.34/person/year, which we agree is an underestimation. For that reason, we re-estimated the cost savings of the SSB tax using a recent obesity cost estimation for Brazil. This cost is closer to what has been estimated in other countries and contains both direct and indirect costs. In the new estimation, instead of using the direct cost of US $10.34/person/year, we are using US$451.3/person/year for direct costs and US$629.2/person/year for indirect costs, which together sums US$1080.5 /person/year. We changed methods, results and discussion to include the new estimation, as follows:

Methods

*Obesity costs were obtained from a 2021 publication which estimated direct and indirect costs attributable to overweight and obesity for eight countries, including Brazil [26]. The total cost of obesity in Brazil was US$ 38.76 billion (16.19 direct and 22.57 indirect costs), using a societal perspective. Direct costs were estimated as the obesity-attributable fraction of healthcare expenditures and indirect costs included economic loss from premature mortality, missed days of work (absenteeism) and reduced productivity while at work (presenteeism) [26]. Using a systematic review that estimated that 87% of the overweight and obesity costs were attributed to obesity, we estimated the obesity costs in Brazil to be US$33.72 billion. In 2018, we estimated a 25.2% obesity prevalence in the adult population in Brazil using POF, resulting in 37.2 million persons with obesity. Dividing the annual cost of obesity by the total number of obesity cases, we estimated an annual cost per capita of US$ 906.92. Using the Consumer Price Index Inflation rate, we converted costs from 2019 to costs in 2023 and obtained an annual cost of US$ 1085.5. Finally, we multiplied the absolute yearly reduction in obesity prevalence by the obesity costs to estimate the total obesity cost savings.*

*Results*

*Figure 2 shows the expected obesity reduction in cases and potential cost savings 10 years after implementing the SSB tax, using own-price elasticities. A 20% tax is expected to reduce the number of people living with obesity by 2.8 million and by 3.7 million with a 30% tax; this would translate into direct cost savings of $US 22.3 billion with a 20% tax and $US 30.0 billion with a 30% tax. Using cross-price elasticities, these estimates increase, so that the reduction in the number of people living in obesity reaches -2.8 million and -3.8 million for a 30% tax; cost savings increase to $US 22.6 billion with a 20%, and $US 30.3 billion with a 30% tax.*

*Discussion*

A SSB tax in Brazil is expected to produce important savings related to obesity prevention. We estimated a reduction of US$ 22.6 billion in obesity costs ten years after the implementation of a 20% SSB tax, US$ 9.4 billion including only direct costs. This 9.4 billion reduction is smaller than what has been estimated in the US (US$ 23.6 billion) but higher than for Mexico (US$ 159.5 million) for a similar tax increase (20%) and a similar period (ten years).[12,33] We compared our direct costs, because in both studies only direct costs were included. Differences could be due to different costs per obesity case, which vary by health system, but also to the number of people with obesity that could be averted (differences in obesity prevalence). Finally, our cost-analysis does not consider the potential benefits derived from the social investment of the tax revenue, particularly if revenue is directed at strengthening obesity prevention measures, which can further increase the health benefits from the tax.

Because the available literature in obesity costs in Brazil is very heterogeneous, our findings on cost reduction also have limitations. A systematic review found that obesity costs in Brazil ranged from USD 133.8 million to USD 6.3 billion per year, (47 times the lowest estimation) [35]. We relied on an international estimation that includes 8 countries, uses a societal perspective, and includes both direct and indirect costs. Since we are using average obesity costs we are not considering that over the 10 years simulated, the Brazilian population will age, which could affect the average cost of obesity; thus, our cost per person could be underestimating the cost at the end of the simulation period.

Discussion

* Line 365: Cost savings as a result of the tax are compared to cases of the US and to Mexico, but no explanation is provided as to why these estimates for Brazil are lower or higher. The study by Long et al. for a US-based tax similarly does not include indirect costs in their cost-effectiveness analysis, so this explanation is less likely to drive differences between the present study and others. Are there other factors in this case, either specific to the costing approach or to the country, that could drive differences in the estimate of cost savings?

Thanks for the observation, we modified the text as follows:

A SSB tax in Brazil is expected to produce important savings related to obesity prevention. We estimated a reduction of US$ 22.6 billion in obesity costs ten years after the implementation of a 20% SSB tax, US$ 9.4 billion including only direct costs. This 9.4 billion reduction is smaller than what has been estimated in the US (US$ 23.6 billion) but higher than for Mexico (US$ 159.5 million) for a similar tax increase (20%) and a similar period (ten years).[12,33] We compared our direct costs, because in both studies only direct costs were included. Differences could be due to discrepancies in cost per obesity case, which vary by health system, but also to the number of people with obesity that could be averted (population size is very different in the three countries). Finally, our cost-analysis does not consider the potential benefits derived from the social investment of the tax revenue, particularly if revenue is directed at strengthening obesity prevention measures, which can further increase the health benefits from the tax.

* Line 369: Greater discussion of this limitation regarding the assumption of constant obesity prevalence over time should be included. Other studies have clearly shown that obesity rates are rising over time, and in fact, the authors state in their introduction that in Brazil the obesity prevalence increased from 11.8% to 20.3% between 2009 and 2019. The use of a static simulation model is a major limitation of the study and should be framed against other published work that employ stochastic approaches to jointly estimate the impact of a tax while considering an individual's own natural weight trajectory.

Thank you for your comment. Stochastic models that consider weight trajectories are applied at a population level, failing to consider important physiological weight change processes and individual-level characteristics and hampering the possibility of SES stratification. Hall’s weight change model has been widely used to model the impact of dietary interventions as it integrates the mechanisms of how changes in energy intake lead to changes in body mass and body composition. We added the following sentence acknowledging this limitation:

*Our estimates depend on a steady-state assumption for body weight, implying a constant obesity prevalence over time; however, the prevalence of obesity in Brazil has increased over time (11.8% to 20.3% between 2009 and 2019). The impact of this assumption depends on the drivers of the obesity trend in Brazil, with the worst-case scenario being an overestimation of the effect that could occur if obesity increases are driven by products not included in the SSB tax. However, SSB are recognized as important drivers of the obesity epidemic in the world. Despite this, future studies should aim at including obesity trends into models.*

Reviewer #4: The study investigates the implications of increasing taxes on sugar-sweetened beverages (SSB) in Brazil, given their link to obesity and related health issues. This study aims to predict the potential outcomes of reintroducing a 20% or 30% excise tax on SSB consumption, obesity rates, and healthcare expenses. Utilizing data from the 2017/2018 Brazilian Household Budget Survey (POF), the researchers examined the price-elasticity of various beverage categories, including SSBs. By applying these elasticities to baseline beverage consumption, the study predicted caloric intake changes, subsequently estimating alterations in weight and obesity rates using a dynamic model. Findings revealed that a 20% tax could lead to a 6.7% decline in obesity prevalence, translating to a reduction of 2.8 million cases and $257.4 million in obesity-related costs over a decade. A 30% tax could result in a 9.1% decrease, equivalent to 3.8 million cases and savings of $345.9 million. The study concludes that revising and implementing such taxes could significantly curb the consumption of unhealthy beverages, combat obesity, and alleviate health-associated costs in Brazil. Overall, this is well-designed and conducted study. The methodologies employed are robust. The findings are especially relevant for policy considerations regarding SSB taxation in Brazil. Below are my specific comments.

1. Line 56-72: In the abstract, it would be beneficial to distinctly separate the methods and findings sections for clarity.

Thank you, separation is requested by the journal.

2. Line 103-106: The data provided about Brazil's consumption and obesity rates is critical. However, the link between SSB consumption and the rise in obesity isn't explicitly stated, and readers might not make the connection. Directly link SSB consumption to the rise in obesity in Brazil for clarity. An added sentence like "This rise in obesity correlates with the high SSB consumption levels seen in the country" might work.

We added that sentence, as recommended:

In 2010, Brazil’s SSB per capita consumption was estimated at 142 ml/day/person.[15] Between 2009 and 2019, the prevalence of obesity in the country increased from 11.8% to 20.3% across all ages, but particularly among young adults,[16] where SSB consumption is more frequent.[15] The rise in obesity has been concurrent with the increase in SSB consumption.[16]

3. Line 126-127: what is a dynamic weight change model? It could be beneficial to provide a brief description of this key method in the method overview. I did not see this method mentioned or described in detail in the following methods section either.

Thanks for the observation. We usually add a first paragraph with a short description of the methods and then we explain in detail each step. We added in the end of this first paragraph a sentence, as follows:

All steps are further explained in the next subsections.

The model description is available in the subsection: **Reduction in body weight, BMI, and obesity prevalence**

*To simulate weight changes attributable to the SSB tax on the Brazilian population, we used a microsimulation approach, a dynamic model developed by Hall et al. [22] The model considers the dynamic physiological adaptations to body weight that lead to changes of resting metabolic rate and physical activity. [22] It has been previously implemented to estimate the impact of the sugar-sweetened beverages tax.[9,23,24] The model considers changes in extracellular fluid, glycogen, and fat and lean tissues caused by the change in caloric consumption but keeping the physical activity constant at the individual level. Bodyweight is the result of the sum of fat mass and fat-free mass, and it is determined by the model using a system of ordinary differential equations. The model is implemented in the bw package in R (*[*https://rdrr.io/github/INSP-RH/bw/*](https://rdrr.io/github/INSP-RH/bw/)*) A detailed description of the equations and model implementation can be found elsewhere.[22] Dietary and anthropometric information from POF were used as inputs for the model to obtain weight changes over ten years, then translated into BMI changes and obesity prevalence changes. An overview of the simulation process is shown in section 3 from S1 Appendix.*

4. Line 162-167: I am curious about the criteria the authors used to select the control variables in the model. Were these variables chosen based on any evidence?

Thank you for the question. The demographic variables are normally associated with household composition (see Blow, 2003), and data on household head (see Preston, 1997). We based our choices on variables used in the papers of the empirical literature. We have complemented the manuscript to clarify such choices.

Blow, Laura. *Demographics in demand systems*. No. 03/18. IFS Working Papers, 2003.

Preston, Ian, Francois Laisney, and Ian A. Crawford. Estimation of household demand systems using unit value data. No. 97-06. ZEW Discussion Papers, 1997.

5. Line 173: when examining the modifying effects of income levels on price elasticities. I noticed the authors omitted income tertile indicators in the model. Typically, when investigating modifying effects of a variable, both the indicators and their interaction terms should be included.

Thank you for seeking clarification on this topic. You are right that we could have included the income tertiles in the model, but instead we used continuous income. We apologize that two important variables that were included in the model were not reported in the former version of the manuscript, which was income and squared income. Continuous income control for much of the effect of income itself in the regressions. To show that, we have compared the estimated elasticities with the revised (including tertiles) and we can see no significant differences.

Table 1 (original)

| Table 1: Price elasticities of SSB by income tertiles, 2018. | | | | | |
| --- | --- | --- | --- | --- | --- |
|  |  | Sugar sweetened beverages | Other beverages | Alcohol beverages | Light/diet beverages |
| Dependent variable: Log of Quantity Acquired (in Liters) | | | | |  |
| Low income | *Mean* | -1.241*** | 0.046*** | -0.122** | -0.174 |
|  | *sd* | -0.015 | -0.017 | -0.053 | -0.124 |
| Middle income | *Mean* | -1.186*** | 0.03 | -0.076 | 0.094 |
|  | *sd* | -0.019 | -0.021 | -0.047 | -0.11 |
| High income | *Mean* | -1.126*** | 0.066*** | -0.141*** | 0.201** |
|  | *sd* | -0.022 | -0.025 | -0.048 | -0.084 |
| *Observations (n)* | | 18,876 | 27,488 | 4,263 | 383 |

Table 1 (original + Income tertiles dummies)

|  |  | Sugar sweetened beverages | Other beverages | Alcohol beverages | Light/diet beverages |
| --- | --- | --- | --- | --- | --- |
| Dependent variable: Log of Quantity Acquired (in Liters) | | | | |  |
| Low income | *Mean* | -1.241*** | 0.051*** | -0.138** | -0.158 |
|  | *sd* | 0.016 | 0.017 | 0.055 | 0.143 |
| Middle income | *Mean* | -1.193*** | 0.023 | -0.090 | 0.122 |
|  | *sd* | 0.019 | 0.021 | 0.047 | 0.111 |
| High income | *Mean* | -1.121*** | 0.068*** | -0.129*** | 0.198** |
|  | *sd* | 0.022 | 0.025 | 0.048 | 0.085 |
| *Observations (n)* | | 18,876 | 27,488 | 4,263 | 383 |

As the results are very similar, and the income variables in levels and squared control for income patterns on average, we have only corrected the paper to include the income variables in the main specification.
