# Supplementary material for: Estimated reduction in obesity prevalence and costs of a 20% and 30% ad valorem excise tax to sugar-sweetened beverages in Brazil: A modeling study
Source: PLoS Med. 2024 Jul 17;21(7):e1004399. doi: 10.1371/journal.pmed.1004399 (PMC11253955; doi:10.1371/journal.pmed.1004399)
Supplement: Attachment — Submitted filename: Response to reviewers.docx [file pmed.1004399.s003.docx]

Dear Dr. Barrientos-Gutierrez,

Thank you very much for re-submitting your manuscript "Expected reduction in obesity prevalence and costs of a 20% and 30% ad valorem excise tax to Sugar-Sweetened-Beverages in Brazil: a modeling study" (PMEDICINE-D-23-02904R2) for review by PLOS Medicine.

I have discussed the paper with my colleagues and the academic editor and it was also seen again by the reviewers. I am pleased to say that provided the remaining comments of reviewer #2 are dealt with we are planning to accept the paper for publication in the journal.

The remaining issues that need to be addressed are listed at the end of this email. Any accompanying reviewer attachments can be seen via the link below. Please take these into account before resubmitting your manuscript:

***Please note while forming your response, if your article is accepted, you may have the opportunity to make the peer review history publicly available. The record will include editor decision letters (with reviews) and your responses to reviewer comments. If eligible, we will contact you to opt in or out.***

In revising the manuscript for further consideration here, please ensure you address the specific points made by each reviewer and the editors. In your rebuttal letter you should indicate your response to the reviewers' and editors' comments and the changes you have made in the manuscript. Please submit a clean version of the paper as the main article file. A version with changes marked must also be uploaded as a marked up manuscript file.

Please also check the guidelines for revised papers at <http://journals.plos.org/plosmedicine/s/revising-your-manuscript> for any that apply to your paper. If you haven't already, we ask that you provide a short, non-technical Author Summary of your research to make findings accessible to a wide audience that includes both scientists and non-scientists. The Author Summary should immediately follow the Abstract in your revised manuscript. This text is subject to editorial change and should be distinct from the scientific abstract.

We expect to receive your revised manuscript within 1 week. Please email us (plosmedicine@plos.org) if you have any questions or concerns.

We ask every co-author listed on the manuscript to fill in a contributing author statement. If any of the co-authors have not filled in the statement, we will remind them to do so when the paper is revised. If all statements are not completed in a timely fashion this could hold up the re-review process. Should there be a problem getting one of your co-authors to fill in a statement we will be in contact. YOU MUST NOT ADD OR REMOVE AUTHORS UNLESS YOU HAVE ALERTED THE EDITOR HANDLING THE MANUSCRIPT TO THE CHANGE AND THEY SPECIFICALLY HAVE AGREED TO IT.

Please ensure that the paper adheres to the PLOS Data Availability Policy (see <http://journals.plos.org/plosmedicine/s/data-availability>), which requires that all data underlying the study's findings be provided in a repository or as Supporting Information. For data residing with a third party, authors are required to provide instructions with contact information for obtaining the data. PLOS journals do not allow statements supported by "data not shown" or "unpublished results." For such statements, authors must provide supporting data or cite public sources that include it.

Please review your reference list to ensure that it is complete and correct. If you have cited papers that have been retracted, please include the rationale for doing so in the manuscript text, or remove these references and replace them with relevant current references. Any changes to the reference list should be mentioned in the rebuttal letter that accompanies your revised manuscript.

Please note, when your manuscript is accepted, an uncorrected proof of your manuscript will be published online ahead of the final version, unless you've already opted out via the online submission form. If, for any reason, you do not want an earlier version of your manuscript published online or are unsure if you have already indicated as such, please let the journal staff know immediately at plosmedicine@plos.org.

If you have any questions in the meantime, please contact me (kjanin@plos.org) or the journal staff (plosmedicine@plos.org).

We look forward to receiving the revised manuscript by Feb 23 2024 11:59PM.

Sincerely,

Katrien Janin, PhD

Senior Editor

PLOS Medicine

[plosmedicine.org](http://plosmedicine.org/)

------------------------------------------------------------

Requests from Editors:

Comments from Reviewers:

Reviewer #1: Dear editor, I thank you for the opportunity to evaluate the second version of the article. Analyzing the document sent by the authors, I realized that the notes I presented were responded to appropriately, and justifications were also presented. Furthermore, considering the notes from the other reviewers, I see that the new version of the article provides more complete information. I have no further notes on the present.

Reviewer #2: The paper has improved, particularly, the perspective and costs are clearer. It did occur to me that there is an issue with the timing of the health care cost and productivity savings that I missed in my previous reading of the paper.

The source study, Okunogbe et al, estimated those costs using a cost-of-illness approach, which aims to take a snapshot of costs in a particular year. Some of these costs are the result of exposures in prior years. The most extreme example is the mortality component of productivity losses, which are calculated by attributing a proportion of deaths to obesity, then multiplying with (productive) life expectancy at the age of death, and with wages. In the model in the present paper, all costs are related to obesity in the year of analysis.

In reality, a change in SSB consumption would translate to a change in body mass (this is covered very well in the model), which would reduce the number of new cases of obesity-related disease (diabetes, heart disease, etc.). Over time, the pool of prevalent cases prevented would rise. Impacts on mortality would follow, and mortality-related productivity gains (and health care costs in added years of life) would trail those. The model used here omits several of these steps, so part of the cost savings are brought forward, potentially by up to decades.

This could be incorporated in the analysis if the approximate timing of the cost savings and productivity gains can be estimated, and the model can be refined to deal with those lagged effects. Alternatively, this could be addressed in the discussion section.

(That does not solve the issue around 'health care costs in added years of life' - see e.g. Van Baal et al, Health Econ. 2007 Apr;16(4):421-33 - which is ignored in cost-of-illness studies too, and best left to future studies that use life table modelling.)

Thank you for the comment, this is a great observation. As mentioned, the time that takes for caloric change to be reflected in obesity is considered by Hall’s weight change model, in which a 50% impact is achieved in the first year, but continues to have an effect until achieving weight equilibrium. For example, for the 20% tax, a total of 2.76 million cases of obesity could be reduced, but in the first year only half of the cases (1.44 million) were reduced (Table D in appendix).

As the reviewer comments, changing obesity does not immediately change disease incidence; it takes time for disease incidence to change to a ‘new equilibrium’. Evidence on time lags, and the shape of change in disease incidence and mortality, following obesity change is limited. We found some information in a multistage life table model (BODE3) about time lags for different diseases. Most of them have a short time lag, ranging from 1 to 5 years (table 8). We cannot differentiate between diseases as the obesity costs in Okunogbe et al include multiple obesity-related diseases. To avoid overestimating cost savings, we assumed no changes for the first three years and the full effects were modeled from the years 4^th^ to 10^th^. Another source of overestimation is not using discount rates. We now added a 5% discount rate to the costs estimation.

We changed methods, results and appendix, as follows:

Methods

The model considers the dynamic physiological adaptations to body weight that lead to changes in resting metabolic rate and physical activity [24] and has been previously implemented to estimate the impact of the sugar-sweetened beverages tax [9,25,26]. It considers changes in extracellular fluid, glycogen, and fat and lean tissues caused by the change in caloric consumption, keeping the physical activity constant. **Thus, the time lag that takes the caloric changes to be reflected in weight is considered by the model.**

Using the Consumer Price Index Inflation rate, we converted costs from 2019 to costs in **2021** and obtained an annual cost **of US$ 942.5. To account for the time lag in morbidity and mortality changes following obesity changes, no cost benefits were estimated during the first three years of intervention, based on average time lags for most obesity-related diseases in a Diet multistage life table.[30]. The benefits of the full effect of the intervention on direct and indirect costs were modeled in years 4–10. To estimate the total cost savings, we multiplied the absolute yearly reduction in obesity prevalence by the direct and indirect obesity costs. We used a 5% discount rate to avoid overestimations [31].** A detailed description of cost estimations is included in section 5 from S1 Appendix.

Results

Figure 2 shows the expected obesity reduction in cases and potential cost savings 10 years after implementing the SSB tax, using own-price elasticities. A 20% tax is expected to reduce the number of people living with obesity by 2.8 million and by 3.7 million with a 30% tax; this would translate into cost savings of $US **13.1 billion** with a 20% tax and $US **17.6 billion** with a 30% tax. Using cross-price elasticities, the reduction in the number of people living in obesity **is similar** with a -2.8 and -3.8 million for a 20% and 30% tax, respectively; cost savings **reaches** **$US 13.3 and $US 17.9 billion** with a 20 and 30% tax, respectively.

We also changed in the overall document: abstract, what this paper add, and discussion to add the new results.

Reviewer #3: Thank you for your close attention to the comments given by the editors and all four reviewers. I have no additional suggestions for revision at this time.

Reviewer #4: In my opinion, the authors have adequately addressed questions and concerns raised by me and other reviewers. I think the paper is acceptable in its current form. Congratulations to the authors!

Any attachments provided with reviews can be seen via the following link:
