# Supplementary material for: Estimated reduction in obesity prevalence and costs of a 20% and 30% ad valorem excise tax to sugar-sweetened beverages in Brazil: A modeling study
Source: PLoS Med. 2024 Jul 17;21(7):e1004399. doi: 10.1371/journal.pmed.1004399 (PMC11253955; doi:10.1371/journal.pmed.1004399)
Supplement: Attachment — Submitted filename: Comments by Reviewer #2 with graphs.docx [file pmed.1004399.s004.docx]

Review of PlosMed SSB tax Brazil v3

The work now contains a lag time between the start of the intervention, and the costs. And a 5% discount rate has been applied. Both are improvements, but the chosen solutions still falls a bit short, I’m afraid.

In reference 30, the BODE3 team wrote, in Table 1: “Time lags for intervention effect: It takes time for a change in body mass index (BMI) to impact on disease incidence. As there are no precise data on just how long these are, we have used wide windows of time lags. For cancers, the time lag is assumed to range between 10 and 30 years. For CHD, stroke, diabetes, and osteoarthritis (the noncancers), the time lag is assumed to be shorter and ranges between 0 and 5 years. Wide uncertainty is included around these estimates.”

That is, the lag time accounts for the impact of a risk factor change on incidence. Cleghorn et al then use a proportional multi-state life table model that accounts for the subsequent lags between incidence and prevalence, and prevalence and mortality, and for the fact that some lost life years are further into the future than others.

To illustrate, I add a few graphs below, where we modelled the potential impact of an SSB tax (compared to BAU) using a model similar to the one used by BODE3. The graphs show the time patterns in IHD, where impact is quick for incidence (we modelled no lag time between exposure change and incidence), slower for prevalence (this builds up over time), and mortality (which lags more as it is a function of prevalence), and HALYs (not visible but this lags even more as it is partly a function of deaths in previous years).

In this paper, you now apply the delay factors intended for incidence, to all those sequelae. (As explained in my previous review, the Okunogbe paper used a cost-of-illness approach, which takes a snapshot of costs in a particular year.) In years 4-10, you use the full impact, which still underestimates the lag. Most of the impact would materialise after that 10-year mark. It would nevertheless be fair to attribute those cost savings in later years to the exposure changes within those first 10 years – it’s just that they would properly need to be discounted in a lifetable or similar structure. Since the benefits would stretch over decades, the impact of discounting would be much greater than in your current calculations, which go up to year 10. In another graph below, you can see that with a 5% discount rate, the ‘net present value’ for an event in year 10 is 61%, but a life year in year 25 would count only for 0.3 life years (NPV).

This would affect mainly the cost of premature mortality, which in Okunogbe’s study “constitute[d] a substantial proportion of indirect costs (about 56%–92%) across all countries”. Although Okunogbe et al ignore this, it would apply also to the direct obesity costs. Longstanding obesity is more costly than recent obesity due to the time it takes to develop disease (see above).

Modelling this properly is clearly not an option here – you would need a sophisticated life table model. The current solution is not elegant and still overestimates the 10-year gains, and this is not explained in the paper; not mentioned in the limitations.

The best solution would be to omit the estimates of the cost savings altogether, and instead focus on the reductions in obesity (by income), which is where the strength of this paper lies. The discussion could then mention the likely impact on obesity-related costs, referring to the Okunogbe paper.

Alternatively, you could proceed as you did now, but with more discussion in the limitations section. You would have to point out the cost savings could be attributed to changes in obesity prevalence in those 10 years, but would in reality materialise largely in the decades following, that discounting would reduce the net present value of the gains (which you accounted for only partially), and that future life table-based modelling could produce more realistic estimates of these impacts over time.

Minor comment: ‘dietary multistate life table’ rather than ‘Diet multistage life table’.
