# Supplementary material for: Estimated reduction in obesity prevalence and costs of a 20% and 30% ad valorem excise tax to sugar-sweetened beverages in Brazil: A modeling study
Source: PLoS Med. 2024 Jul 17;21(7):e1004399. doi: 10.1371/journal.pmed.1004399 (PMC11253955; doi:10.1371/journal.pmed.1004399)
Supplement: Attachment — Submitted filename: Brazil SSB tax plos med 3rd review.docx [file pmed.1004399.s005.docx]

Dear Dr. Barrientos-Gutierrez,

Thank you very much for re-submitting your manuscript "Expected reduction in obesity prevalence and costs of a 20% and 30% ad valorem excise tax to Sugar-Sweetened-Beverages in Brazil: a modeling study" (PMEDICINE-D-23-02904R3) for review by PLOS Medicine.

I have discussed the paper with my colleagues and the academic editor and it was also seen again by the reviewer. I am pleased to say that provided the remaining editorial and production issues are dealt with we are planning to accept the paper for publication in the journal.

The remaining issues that need to be addressed are listed at the end of this email. Any accompanying reviewer attachments can be seen via the link below. Please take these into account before resubmitting your manuscript:

********

***Please note while forming your response, if your article is accepted, you may have the opportunity to make the peer review history publicly available. The record will include editor decision letters (with reviews) and your responses to reviewer comments. If eligible, we will contact you to opt in or out.***

In revising the manuscript for further consideration here, please ensure you address the specific points made by each reviewer and the editors. In your rebuttal letter you should indicate your response to the reviewers' and editors' comments and the changes you have made in the manuscript. Please submit a clean version of the paper as the main article file. A version with changes marked must also be uploaded as a marked up manuscript file.

Please also check the guidelines for revised papers at http://journals.plos.org/plosmedicine/s/revising-your-manuscript for any that apply to your paper. If you haven't already, we ask that you provide a short, non-technical Author Summary of your research to make findings accessible to a wide audience that includes both scientists and non-scientists. The Author Summary should immediately follow the Abstract in your revised manuscript. This text is subject to editorial change and should be distinct from the scientific abstract.

We expect to receive your revised manuscript within 1 week. Please email us (plosmedicine@plos.org) if you have any questions or concerns.

We ask every co-author listed on the manuscript to fill in a contributing author statement. If any of the co-authors have not filled in the statement, we will remind them to do so when the paper is revised. If all statements are not completed in a timely fashion this could hold up the re-review process. Should there be a problem getting one of your co-authors to fill in a statement we will be in contact. YOU MUST NOT ADD OR REMOVE AUTHORS UNLESS YOU HAVE ALERTED THE EDITOR HANDLING THE MANUSCRIPT TO THE CHANGE AND THEY SPECIFICALLY HAVE AGREED TO IT.

Please ensure that the paper adheres to the PLOS Data Availability Policy (see http://journals.plos.org/plosmedicine/s/data-availability), which requires that all data underlying the study's findings be provided in a repository or as Supporting Information. For data residing with a third party, authors are required to provide instructions with contact information for obtaining the data. PLOS journals do not allow statements supported by "data not shown" or "unpublished results." For such statements, authors must provide supporting data or cite public sources that include it.

To enhance the reproducibility of your results, we recommend that you deposit your laboratory protocols in protocols.io, where a protocol can be assigned its own identifier (DOI) such that it can be cited independently in the future. Additionally, PLOS ONE offers an option to publish peer-reviewed clinical study protocols. Read more information on sharing protocols at https://plos.org/protocols?utm_medium=editorial-email&utm_source=authorletters&utm_campaign=protocols

Please review your reference list to ensure that it is complete and correct. If you have cited papers that have been retracted, please include the rationale for doing so in the manuscript text, or remove these references and replace them with relevant current references. Any changes to the reference list should be mentioned in the rebuttal letter that accompanies your revised manuscript.

Please note, when your manuscript is accepted, an uncorrected proof of your manuscript will be published online ahead of the final version, unless you've already opted out via the online submission form. If, for any reason, you do not want an earlier version of your manuscript published online or are unsure if you have already indicated as such, please let the journal staff know immediately at plosmedicine@plos.org.

If you have any questions in the meantime, please contact me or the journal staff on plosmedicine@plos.org.

We look forward to receiving the revised manuscript by Mar 21 2024 11:59PM.

Sincerely,

Katrien Janin, PhD

Senior Editor

PLOS Medicine

plosmedicine.org

------------------------------------------------------------

Comments from Reviewers:

Reviewer #2: The work now contains a lag time between the start of the intervention, and the costs. And a 5% discount rate has been applied. Both are improvements, but the chosen solutions still falls a bit short, I'm afraid.

In reference 30, the BODE3 team wrote, in Table 1: "Time lags for intervention effect: It takes time for a change in body mass index (BMI) to impact on disease incidence. As there are no precise data on just how long these are, we have used wide windows of time lags. For cancers, the time lag is assumed to range between 10 and 30 years. For CHD, stroke, diabetes, and osteoarthritis (the noncancers), the time lag is assumed to be shorter and ranges between 0 and 5 years. Wide uncertainty is included around these estimates."

That is, the lag time accounts for the impact of a risk factor change on incidence. Cleghorn et al then use a proportional multi-state life table model that accounts for the subsequent lags between incidence and prevalence, and prevalence and mortality, and for the fact that some lost life years are further into the future than others.

To illustrate, I had hoped to add a few graphs. The system will not allow that, but I'll see if the editorial team can help. The graphs show the results of a study in which we modelled the potential impact of an SSB tax (compared to BAU) using a model similar to the one used by BODE3. The graphs show the time patterns in IHD, where impact is quick for incidence (we modelled no lag time between exposure change and incidence), slower for prevalence (this builds up over time), and mortality (which lags more as it is a function of prevalence), and HALYs (not visible but this lags even more as it is partly a function of deaths in previous years).

In this paper, you now apply the delay factors intended for incidence, to all those sequelae. (As explained in my previous review, the Okunogbe paper used a cost-of-illness approach, which takes a snapshot of costs in a particular year.) In years 4-10, you use the full impact, which still underestimates the lag. Most of the impact would materialise after that 10-year mark. It would nevertheless be fair to attribute those cost savings in later years to the exposure changes within those first 10 years - it's just that they would properly need to be discounted in a lifetable or similar structure. Since the benefits would stretch over decades, the impact of discounting would be much greater than in your current calculations, which go up to year 10. In another graph below, you can see that with a 5% discount rate, the 'net present value' for an event in year 10 is 61%, but a life year in year 25 would count only for 0.3 life years (NPV).

This would affect mainly the cost of premature mortality, which in Okunogbe's study "constitute[d] a substantial proportion of indirect costs (about 56%-92%) across all countries". Although Okunogbe et al ignore this, it would apply also, to a lesser extent, to the direct obesity costs. Longstanding obesity is more costly than recent obesity due to the time it takes to develop disease (see above).

Modelling this properly is clearly not an option here - you would need a sophisticated life table model. The current solution is not elegant and still overestimates the 10-year gains, and this is not explained in the paper; not mentioned in the limitations.

The best solution would be to omit the estimates of the cost savings altogether, and instead focus on the reductions in obesity (by income), which is where the strength of this paper lies. The discussion could then mention the likely impact on obesity-related costs, referring to the Okunogbe paper.

Alternatively, you could proceed as you did now, but with more discussion in the limitations section. You would have to point out the cost savings could be attributed to changes in obesity prevalence in those 10 years, but would in reality materialise largely in the decades following, that discounting would reduce the net present value of the gains (which you accounted for only partially), and that future life table-based modelling could produce more realistic estimates of these impacts over time.

R. We truly appreciate the time you have spent revising our manuscript, we have learned and reflected a lot through your comments: thank you. We concur that our model is ill-suited to include a precise estimate of cost savings. The editorial team recommended to follow your second option, so we added the following to limitations:

Our model could overestimate cost savings. Prevalent costs of obesity were obtained from Okunogbe, et al., [28], yet, the obesity changes estimated in our model will take years to fully materialize after the intervention. Some indicators will improve fast after weight loss, such as disease incidence, but others will take years to decades to change, such as mortality. To fully model these dynamics, we would have needed to use a life-table model with an open population to capture these changes, discount the health gains over time, and go beyond the 10-year modeling timeframe used in our analysis. We attempted to reduce overestimation by introducing a 3 year-lag of no cost savings and including a 5% discount rate, but this could still fall short. Future studies interested in modeling obesity cost savings should rely on life-table models to capture the net present value of health gains. Also, the literature on obesity costs in Brazil provides heterogeneous figures. A systematic review found that obesity costs in Brazil ranged from USD 133.8 million to USD 6.3 billion per year (47 times the lowest estimation) [40]. We relied on an international estimation that includes 8 countries uses a societal perspective, and both direct and indirect costs. We are not considering that over the 10 years simulated, the Brazilian population will age, which could affect the average cost of obesity; our cost per person could underestimate the cost at the end of the simulation period. Our estimates depend on a steady-state assumption for body weight, implying a constant obesity prevalence over time; however, the prevalence of obesity in Brazil has increased over time (11.8% to 20.3% between 2009 and 2019). The impact of this assumption depends on the drivers of the obesity trend in Brazil, with the worst-case scenario being an overestimation of the effect that could occur if obesity increases are driven by products not included in the SSB tax. However, SSBs are recognized as important drivers of the obesity epidemic in the world. Despite this, future studies should aim at including obesity trends into models. Finally, we simulated this intervention in the adult population, so further reductions in obesity prevalence are to be expected in children and adolescents, who may change dietary habits and reduce weight as they transition into adulthood.

Minor comment: 'dietary multistate life table' rather than 'Diet multistage life table'.

We changed to dietary multistate life table as suggested.

------------------------------------------------------------

Requests from Editors:

After a long discussion with my editorial colleagues, and taken fully on board the reviewer comments, we like to suggest the following solution to move forward:

We prefer to go down the route to ask you to expanded the limitations in the discussion and ask to use very appropriate language when talking about the projected cost savings in your manuscript. The reason we prefer to go down that route is that the current calculation do give an indication of the cost involved but we agree with the reviewer's points about time lag and the resulting likelihood that their cost savings estimates are inflated MUST be discussed carefully in the discussion/limitations. We therefore ask you to implement the alternative option as presented by the reviewer.

We like to thank the reviewer for such a terrific and informative discussion

Please do not hesitate to contact me directly at kjanin@plos.org if you have any question about the above.

Thank you for the opportunity to revise the discussion and expand the limitation section about the cost savings estimation.
